# Classification of soft decision-making methods via fuzzy parameterized fuzzy soft matrices and their performance-based statistical analysis in machine learning

**DOI:** 10.1371/journal.pone.0348760

**Published:** 2026-05-13

**Authors:** Ömer Karakoç, Samet Memiş, Bahar Sennaroglu

**Affiliations:** 1 Department of Industrial Engineering, Marmara University, İstanbul, Türkiye; 2 Department of Industrial Engineering, Altınbaş University, İstanbul, Türkiye; 3 Department of Marine Engineering, Bandırma Onyedi Eylül University, Balıkesir, Türkiye; National Institute of Technology, India (Institute of National Importance), INDIA

## Abstract

This study provides a comprehensive evaluation and classification of 35 soft decision-making (SDM) algorithms based on fuzzy parameterized fuzzy soft matrices (*fpfs*-matrices). Although *fpfs*-matrices offer a strong mathematical framework for modeling uncertainty, there has been a lack of large-scale comparisons of their derivative SDM methods in machine learning. To address this, we used the Comparison Matrix-Based Fuzzy Parameterized Fuzzy Soft Classifier (FPFS-CMC) to benchmark these 35 algorithms across ten diverse datasets from the UCI Machine Learning Repository. The methods were thoroughly assessed utilizing metrics such as accuracy, precision, recall (sensitivity), specificity, and F1-score, and statistical significance was confirmed using the Friedman and Nemenyi tests. Our results show that SDM methods via *fpfs*-matrices perform competitively in classification tasks involving uncertainty. Notably, the best algorithms according to F1-scores were A19 (Rank 1), YHX14 (Rank 2), and a three-way tie for Rank 3 among VMH16, AKO18o, and A19/2. By identifying the most effective algorithms and offering a structured decision-support framework, this research provides both a theoretical reference and practical guidance for practitioners selecting SDM methods for complex machine learning challenges.

## 1. Introduction

Traditional mathematical methods may not be sufficient when dealing with problems that involve uncertainties. To overcome this issue, fuzzy sets [1] and soft sets [2] have been introduced to address uncertainty and applied in various fields. Various extensions of fuzzy and soft sets have emerged, including fuzzy soft sets [3,4], fuzzy parameterized soft sets [5], and fuzzy parameterized fuzzy soft sets (*fpfs*-sets) [6]. The concept of *fpfs*-sets has been particularly prominent due to its modeling ability. However, when dealing with a problem that has several criteria and high uncertainty, computerizing the *fpfs*-sets becomes necessary. To that end, soft matrices [7], fuzzy soft matrices [8] and fuzzy parameterized fuzzy soft matrices (*fpfs*-matrices) [9] have been proposed. *fpfs*-matrices have a broad scope by combining matrices, fuzzy and soft sets, and their extension. These provide the ability to address various types of uncertainty. Therefore, some soft decision-making (SDM) algorithms constructed by soft sets, fuzzy soft sets, fuzzy parameterized soft sets, *fpfs*-sets, soft matrices, and fuzzy soft matrices have been configured via *fpfs*-matrices. Since *fpfs*-matrices possess a modular structure and fuzzy criteria, they can be implemented more efficiently in applications. Recently, *fpfs*-matrices have been used in various decision-making problems, such as recruitment scenarios [10–12] and performance-based value assignment (PVA) problems in image denoising [13–16] as well as classification problems in machine learning [17–20].

Compared to algebraic research on new operations of soft sets [21], the potential applications of soft sets have been discussed in detail in various studies, and it has been stated that integrating soft sets into decision-making processes improves uncertainty management thanks to their flexible and parametric structures [22, 23]. In addition, the increasing importance of soft set theory in multi-criteria decision problems has been emphasized in a systematic review [24]. In this context, *fpfs*-matrices offer more detailed and adaptable analyses by integrating fuzziness and parametric structure compared to classical matrix models, and produce meaningful and consistent results, especially in multi-criteria decision support systems. In addition, SDM methods based on *fpfs*-matrices and *fpfs*-matrices-based *k*-nearest neighbor classifiers, such as FPFS-kNN [25], have been applied to energy efficiency analysis, evaluating heavy commercial vehicles in the logistics sector, energy planning in Türkiye, and academic performance evaluation. Aside from the studies mentioned above, the optimal feature selection-based dental caries prediction study [26] and the research demonstrating that *fpfs*-based machine learning algorithms effectively predict NO_x_ emissions in the maritime field [27] have significantly inspired the application of this method across various disciplines. Following these developments, *ifpifs*-matrices structures, which offer a higher degree of uncertainty modeling capacity, have been developed, enhancing intuitiveness in decision-making. The studies [28,29] have showcased the advantages of this structure in applications such as evaluation and filter selection using multiple *ifpifs*-matrices. One of the most recent contributions is pioneering the development of next-generation algorithms for artificial intelligence applications by integrating the *ifpifs*-matrices-based SDM approach into adaptive machine learning models [30]. All these studies demonstrate that SDM methods address a broad range of applications when utilized with *fpfs*-matrices and *ifpifs*-matrices, providing an effective tool for modeling high degrees of uncertainty. The scope of this work is confined to *fpfs*-matrices due to their ease of implementation.

Although a comparison is provided, no performance metrics are provided to rank the algorithms’ success. Five test cases have been defined to eliminate the unsuccessful ones. These include five scenarios where an expert can naturally rank alternatives as the basis for the test cases [31]. An SDM technique completes a test case by generating its specified ranking order. To evaluate and compare the performance of algorithms that pass the test, one can use performance metrics and assess them with different machine learning algorithms. Latterly, SDM methods via *fpfs*-matrices have been applied to machine learning in a very striking way. The machine learning algorithms called FPFS-CMC [32] and FPFS-AC [33] use two SDM approaches. Despite advancements and successful implementations of various SDM algorithms in specific areas, a major gap persists in the literature: there is no unified framework for comparing their modeling capabilities and performance limits. Current research mainly uses isolated SDM techniques on narrow case studies, making objective benchmarking under consistent conditions difficult. As a result, researchers and practitioners lack a systematic framework for determining which SDM approach is best for different decision-making scenarios or high-dimensional data environments.

To address this issue, this study introduces a modular evaluation framework that combines SDM methods within machine learning architecture. Using the Comparison Matrix-Based Fuzzy Parameterized Fuzzy Soft Classifier (FPFS-CMC) as a standardized benchmarking tool, we provide a quantitative and statistically supported assessment of SDM performance. The main goal of this paper is to systematically classify 35 prominent SDM algorithms and perform an extensive comparative analysis across 10 real-world datasets from the UCI Machine Learning Repository. The key contributions of this research are fourfold:

1. This paper offers the first comprehensive taxonomy and comparison of 35 SDM algorithms from the literature.
2. By embedding SDM components into the modular FPFS-CMC structure, it converts qualitative decision-making tools into measurable machine learning classifiers.
3. This study provides an objective ranking of SDM methods based on multiple performance metrics (accuracy, precision, recall, specificity, and F1-score), validated through rigorous statistical tests.
4. It presents a decision-support framework that connects the abstract theory of *fpfs*-matrices with real-world data classification problems.

Considering these points, this paper introduces a significant methodological shift from “single case” applications to a “comprehensive” performance evaluation. By benchmarking 35 different SDM algorithms across ten real-world datasets, we provide a statistically validated reference for uncertainty modeling in artificial intelligence. This study highlights the most effective algorithms, such as A19 and YHX14, and creates a modular framework for future advancements in soft decision-making.

The organization of this paper is as follows: The second section introduces the concepts of *fpfs*-sets and *fpfs*-matrices, along with some related properties. The third section presents five test cases for *fpfs*-matrices and compares SDM methods across them. The fourth section provides a brief introduction to structured *fpfs*-matrices, their application fields, decision models, and the mathematical operators used. The fifth section compares and classifies algorithms based on their F1-scores. The performance changes of algorithms are discussed through statistical analysis. The sixth section analyzes the computational complexities of the SDM methods. The last section outlines the results and suggests directions for future work.

## 2. Materials & Methods

This section presents the concepts of *fpfs*-sets [6] and *fpfs*-matrices [9]. From now on, let E be a parameter set, F(E) be the set of all the fuzzy sets over E, and μ∈F(E). Here, a fuzzy set is denoted by { μ(x)x | x∈E}.

Definition 1.[6] Let U be a universal set, μ∈F(E), and α be a function from μ to F(U). Then, the set {( μ(x)x,α( μ(x)x))| x∈E}, being the graphic of α, is called a fuzzy parameterized fuzzy soft set (*fpfs*-set) parameterized via E over U (or briefly over U).

Across the present paper, the set of all the *fpfs*-sets over U is denoted by $FPFS_{E}(U)$. In $FPFS_{E}(U)$, since the graph(α) and α generate each other uniquely, the notations are interchangeable. Thus, as long as it leads to no confusion, α stands for an *fpfs*-set graph(α).

Example 1. Let $E=\left\{x_{\text{1}},x_{\text{2}},x_{\text{3}}\right\}$ and $U=\left\{u_{\text{1}},u_{\text{2}},u_{\text{3}},u_{\text{4}}\right\}$. Then,


$$\begin{array}{c} \alpha =\left\{\left(\;\text{0}.\text{3}x_{\text{1}},\left\{\;\text{0}.\text{4}u_{\text{2}},\;\text{0}.\text{3}u_{\text{3}},\;\text{0}.\text{6}u_{\text{4}}\right\}\right),\left(\;\text{0}.\text{2}x_{\text{2}},\left\{\;\text{1}u_{\text{1}},\;\text{0}.\text{4}u_{\text{2}},\;\text{0}.\text{2}u_{\text{3}},\;\text{0}.\text{3}u_{\text{4}}\right\}\right),\left(\;\text{0}.\text{4}x_{\text{3}},\left\{\;\text{0}.\text{4}u_{\text{1}},\;\text{0}.\text{2}u_{\text{3}},\;\text{0}.\text{5}u_{\text{4}}\right\}\right)\right\} \end{array}$$

is an *fpfs*-set over U.

Definition 2.[9] Let $\alpha \in FPFS_{E}(U)$. Then, $\left[a_{ij}\right]$ is called *fpfs*-matrix of α and is defined by


$$\begin{array}{c} \left[a_{ij}\right]=[\begin{array}{ccccc} a_{\text{0}\text{1}} & a_{\text{0}\text{2}} & a_{\text{0}\text{3}} & \ldots & a_{\text{0}n}\\ a_{\text{1}\text{1}} & a_{\text{1}\text{2}} & a_{\text{1}\text{3}} & \ldots & a_{\text{1}n}\\ \vdots & \vdots & \vdots & \ddots & \vdots \\ a_{m\text{1}} & a_{m\text{2}} & a_{m\text{3}} & \ldots & a_{mn} \end{array}] \end{array}$$

such that for i∈{0,1,2, ⋯, m} and j∈{1,2,⋯, n}*,*


$$\begin{array}{c} a_{ij}:=\left\{ \begin{array}{c} @l\mu \left(x_{j}\right),\;i=\text{0}\\ \alpha \left(\;\mu \left(x_{j}\right)x_{j}\right)\left(u_{i}\right),\;i\neq \text{0} \end{array}\right.\; \end{array}$$

Here, if |U|=m−1 and |E|=n, then $\left[a_{ij}\right]$ has order m×n.

Hereinafter, the set of all the *fpfs*-matrices parameterized via E over U is denoted by $FPFS_{E}[U]$.

Example 2.The *fpfs*-matrix of α provided in Example 1 is as follows:


$$\begin{array}{c} \left[a_{ij}\right]=[\begin{array}{ccc} \text{0}.\text{3} & \text{0}.\text{2} & \text{0}.\text{4}\\ \text{0} & \text{1} & \text{0}.\text{4}\\ \text{0}.\text{4} & \text{0}.\text{4} & \text{0}\\ \text{0}.\text{3} & \text{0}.\text{2} & \text{0}.\text{2}\\ \text{0}.\text{6} & \text{0}.\text{3} & \text{0}.\text{5} \end{array}] \end{array}$$

Definition 3.Let $\left[a_{ij}\right]\in M_{m\times n}\left(R\right)$. Then, feature fuzzification of $\left[a_{ij}\right]$ is defined by


$$\begin{array}{c} {\tilde{a}}_{ij}\;:=\left\{ \begin{array}{cc} \frac{a_{ij}}{\underset{k}{\text{max}}a_{kj}}, & \underset{k}{\text{max}}s_{k\text{1}}\neq \text{0}\\ \text{1}, & \underset{k}{\text{max}}s_{k\text{1}}=\text{0} \end{array}\right.\; \end{array}$$

Definition 4.Let $\left[s_{i\text{1}}\right]\in M_{(m-\text{1})\times \text{1}}\left(R\right)$ such that m≥2. Then, normalization $\left[{\hat{s}}_{i\text{1}}\right]$ of $\left[s_{i\text{1}}\right]$ is defined by


$$\begin{array}{c} {\hat{s}}_{i\text{1}}\;:=\left\{ \begin{array}{cc} \frac{s_{i\text{1}}-\underset{k}{\text{min}}s_{k\text{1}}}{\underset{k}{\text{max}}s_{k\text{1}}-\underset{k}{\text{min}}s_{k\text{1}}}, & \underset{k}{\text{max}}s_{k\text{1}}\neq \underset{k}{\text{min}}s_{k\text{1}}\\ \text{1}, & \underset{k}{\text{max}}s_{k\text{1}}=\underset{k}{\text{min}}s_{k\text{1}} \end{array}\right.\; \end{array}$$

Table 1 presents a list of 35 SDM algorithms [31,34–36], along with their abbreviated versions. For more details about the algorithms, see their references in Table 1.

**Table 1:** The list of 35 algorithms to be classified and their abbreviations.

No	Algorithm Abbreviation	References	No	Algorithm Abbreviation	References
**1**	CXL13(λ)	[31,37]	**19**	AKO18a	[34,38]
**2**	WQ14(κ)	[31,39]	**20**	AKO18o	[34,38]
**3**	YHX14(α,β)	[31,40]	**21**	RH18	[34,41]
**4**	DC15(α)	[31,42]	**22**	SM13(w,α)	[31,43]
**5**	ZZ19(λ,γ)	[34,44]	**23**	NKY17	[31,45]
**6**	CEC11	[4,36]	**24**	Z14/2	[31,46]
**7**	MBR01	[3,35]	**25**	MR13	[31,47]
**8**	G17(R)	[34,48]	**26**	MR13/2	[31,47]
**9**	LQP17(w)	[34,49]	**27**	RS16	[31,50]
**10**	KM11(𝐼𝑛))	[36,51]	**28**	AT18(λ)	[34,52]
**11**	LL18 (λ)	[34,53]	**29**	P18	[34,54]
**12**	A19	[34,55]	**30**	A19/2(R)	[34,55]
**13**	CXL13/2(λ)	[34,37]	**31**	SS19/2	[34,56]
**14**	HG13	[34,57]	**32**	SS19/3	[34,56]
**15**	MRB02	[35,58]	**33**	SS19/4	[34,56]
**16**	ZXZ15(α)	[34,59]	**34**	SS19/5(w)	[32,60]
**17**	VMH16	[34,61]	**35**	ZCW19(δ,θ)	[32,62]
**18**	RH17(α)	[34,63]			

## 3. Test Cases for the Comparison of the SDM Methods

This section outlines five test cases from [31] to compare the decision-making performance of SDM methods that use single, double, or multiple *fpfs*-matrices. Therefore, each test case consists of t *fpfs*-matrices $\left[\overset{\text{1}}{\underset{ij}{a}}\right],\;\left[\overset{\text{2}}{\underset{ij}{a}}\right],\ldots ,\;\left[\overset{t}{\underset{ij}{a}}\right]$, which has order m×n and manifests the same ranking order of alternatives without employing SDM methods. If an SDM method utilizes a single *fpfs*-matrix, we only use $\left[\overset{\text{1}}{\underset{ij}{a}}\right]$. Similarly, if double, we use $\left[\overset{\text{1}}{\underset{ij}{a}}\right]$ and $\left[\overset{\text{2}}{\underset{ij}{a}}\right]$. If an SDM method produces the ranking order provided in a test case, it is said to accomplish the test case. In this section, let t=3, m=5, n=4, $U=\left\{u_{\text{1}},u_{\text{2}},u_{\text{3}},u_{\text{4}}\right\}$ be the set of alternatives, and $E=\left\{x_{\text{1}},x_{\text{2}},x_{\text{3}},x_{\text{4}}\right\}$ be the set of parameters.

### 3.1. Test Case 1

Test Case 1 constructs three *fpfs*-matrices ${\left[\overset{\text{1}}{\underset{ij}{a}}\right]}_{\text{5}\times \text{4}}$, ${\left[\overset{\text{2}}{\underset{ij}{a}}\right]}_{\text{5}\times \text{4}}$, and ${\left[\overset{\text{3}}{\underset{ij}{a}}\right]}_{\text{5}\times \text{4}}$ such that for all $\;j\in I_{\text{4}}$ and $k\in I_{\text{3}}$, $\overset{k}{\underset{\text{0}\text{1}}{a}}=\overset{k}{\underset{\text{0}\text{2}}{a}}=\overset{k}{\underset{\text{0}\text{3}}{a}}=\overset{k}{\underset{\text{0}\text{4}}{a}}$ and $\overset{k}{\underset{\text{1}j}{a}}≺\overset{k}{\underset{\text{2}j}{a}}≺\overset{k}{\underset{\text{3}j}{a}}≺\overset{k}{\underset{\text{4}j}{a}}$. Therefore, $\overset{k}{\underset{\text{0}j}{a}}\overset{k}{\underset{\text{1}j}{a}}≺\overset{k}{\underset{\text{0}j}{a}}\overset{k}{\underset{\text{2}j}{a}}≺\overset{k}{\underset{\text{0}j}{a}}\overset{k}{\underset{\text{3}j}{a}}≺\overset{k}{\underset{\text{0}j}{a}}\overset{k}{\underset{\text{4}j}{a}}$, for all$\;j\in I_{\text{4}}$ and $k\in I_{\text{3}}$. For each *fpfs*-matrix herein, the ranking order of alternatives is $u_{\text{1}}≺u_{\text{2}}≺u_{\text{3}}≺u_{\text{4}}$. For example,


$$\begin{array}{c} \left[\overset{\text{1}}{\underset{ij}{a}}\right]:=[\begin{array}{cccc} \text{0}.\text{40} & \text{0}.\text{40} & \text{0}.\text{40} & \text{0}.\text{40}\\ \text{0}.\text{7}\text{0} & \text{0}.\text{6}\text{0} & \text{0}.\text{5}\text{0} & \text{0}.\text{4}\text{0}\\ \text{0}.\text{80} & \text{0}.\text{70} & \text{0}.\text{6}\text{0} & \text{0}.\text{5}\text{0}\\ \text{0}.\text{90} & \text{0}.\text{80} & \text{0}.\text{7}\text{0} & \text{0}.\text{6}\text{0}\\ \text{1} & \text{0}.\text{9}\text{0} & \text{0}.\text{8}\text{0} & \text{0}.\text{7}\text{0} \end{array}],\;\left[\overset{\text{2}}{\underset{ij}{a}}\right]:=[\begin{array}{cccc} \text{0}.\text{40} & \text{0}.\text{40} & \text{0}.\text{40} & \text{0}.\text{40}\\ \text{0}.\text{3}\text{0} & \text{0}.\text{2}\text{0} & \text{0}.\text{1}\text{0} & \text{0}\\ \text{0}.\text{40} & \text{0}.\text{30} & \text{0}.\text{2}\text{0} & \text{0}.\text{1}\text{0}\\ \text{0}.\text{50} & \text{0}.\text{40} & \text{0}.\text{3}\text{0} & \text{0}.\text{2}\text{0}\\ \text{0}.\text{6}\text{0} & \text{0}.\text{5}\text{0} & \text{0}.\text{4}\text{0} & \text{0}.\text{3}\text{0} \end{array}],\text{ and }\left[\overset{\text{3}}{\underset{ij}{a}}\right]:=[\begin{array}{cccc} \text{0}.\text{40} & \text{0}.\text{40} & \text{0}.\text{40} & \text{0}.\text{40}\\ \text{0}.\text{5}\text{0} & \text{0}.\text{4}\text{0} & \text{0}.\text{3}\text{0} & \text{0}.\text{2}\text{0}\\ \text{0}.\text{60} & \text{0}.\text{50} & \text{0}.\text{4}\text{0} & \text{0}.\text{3}\text{0}\\ \text{0}.\text{70} & \text{0}.\text{60} & \text{0}.\text{5}\text{0} & \text{0}.\text{4}\text{0}\\ \text{0}.\text{8}\text{0} & \text{0}.\text{7} & \text{0}.\text{6}\text{0} & \text{0}.\text{5}\text{0} \end{array}] \end{array}$$

### 3.2. Test Case 2

Test Case 2 constructs three *fpfs*-matrices ${\left[\overset{\text{1}}{\underset{ij}{b}}\right]}_{\text{5}\times \text{4}}$, ${\left[\overset{\text{2}}{\underset{ij}{b}}\right]}_{\text{5}\times \text{4}}$, and ${\left[\overset{\text{3}}{\underset{ij}{b}}\right]}_{\text{5}\times \text{4}}$ such that for all$\;j\in I_{\text{4}}$ and $k\in I_{\text{3}}$, $\overset{k}{\underset{\text{0}\text{1}}{b}}=\overset{k}{\underset{\text{0}\text{2}}{b}}=\overset{k}{\underset{\text{0}\text{3}}{b}}=\overset{k}{\underset{\text{0}\text{4}}{b}}$ and $\overset{k}{\underset{\text{4}j}{b}}≺\overset{k}{\underset{\text{3}j}{b}}≺\overset{k}{\underset{\text{2}j}{b}}≺\overset{k}{\underset{\text{1}j}{b}}$. Therefore, $\overset{k}{\underset{\text{0}j}{b}}\overset{k}{\underset{\text{4}j}{b}}≺\overset{k}{\underset{\text{0}j}{b}}\overset{k}{\underset{\text{3}j}{b}}≺\overset{k}{\underset{\text{0}j}{b}}\overset{k}{\underset{\text{2}j}{b}}≺\overset{k}{\underset{\text{0}j}{b}}\overset{k}{\underset{\text{1}j}{b}}$, for all$\;j\in I_{\text{4}}$ and $k\in I_{\text{3}}$. For each *fpfs*-matrix herein, the ranking order of alternatives is $u_{\text{4}}≺u_{\text{3}}≺u_{\text{2}}≺u_{\text{1}}$. For example,


$$\begin{array}{c} \left[\overset{\text{1}}{\underset{ij}{b}}\right]:=[\begin{array}{cccc} \text{0}.\text{70} & \text{0}.\text{70} & \text{0}.\text{70} & \text{0}.\text{70}\\ \text{1} & \text{0}.\text{9}\text{0} & \text{0}.\text{8}\text{0} & \text{0}.\text{7}\text{0}\\ \text{0}.\text{90} & \text{0}.\text{80} & \text{0}.\text{7}\text{0} & \text{0}.\text{6}\text{0}\\ \text{0}.\text{80} & \text{0}.\text{70} & \text{0}.\text{6}\text{0} & \text{0}.\text{5}\text{0}\\ \text{0}.\text{7}\text{0} & \text{0}.\text{6}\text{0} & \text{0}.\text{5}\text{0} & \text{0}.\text{4}\text{0} \end{array}],\;\left[\overset{\text{2}}{\underset{ij}{b}}\right]:=[\begin{array}{cccc} \text{0}.\text{70} & \text{0}.\text{70} & \text{0}.\text{70} & \text{0}.\text{70}\\ \text{0}.\text{6}\text{0} & \text{0}.\text{5}\text{0} & \text{0}.\text{4}\text{0} & \text{0}.\text{3}\text{0}\\ \text{0}.\text{50} & \text{0}.\text{40} & \text{0}.\text{3}\text{0} & \text{0}.\text{2}\text{0}\\ \text{0}.\text{40} & \text{0}.\text{30} & \text{0}.\text{2}\text{0} & \text{0}.\text{1}\text{0}\\ \text{0}.\text{3}\text{0} & \text{0}.\text{2}\text{0} & \text{0}.\text{1}\text{0} & \text{0} \end{array}],\text{ and }\left[\overset{\text{3}}{\underset{ij}{b}}\right]:=[\begin{array}{cccc} \text{0}.\text{70} & \text{0}.\text{70} & \text{0}.\text{70} & \text{0}.\text{70}\\ \text{0}.\text{8}\text{0} & \text{0}.\text{7}\text{0} & \text{0}.\text{6}\text{0} & \text{0}.\text{5}\text{0}\\ \text{0}.\text{70} & \text{0}.\text{60} & \text{0}.\text{5}\text{0} & \text{0}.\text{4}\text{0}\\ \text{0}.\text{60} & \text{0}.\text{50} & \text{0}.\text{4}\text{0} & \text{0}.\text{3}\text{0}\\ \text{0}.\text{5}\text{0} & \text{0}.\text{4}\text{0} & \text{0}.\text{3}\text{0} & \text{0}.\text{2}\text{0} \end{array}] \end{array}$$

### 3.3. Test Case 3

Test Case 3 constructs three *fpfs*-matrices ${\left[\overset{\text{1}}{\underset{ij}{c}}\right]}_{\text{5}\times \text{4}}$, ${\left[\overset{\text{2}}{\underset{ij}{c}}\right]}_{\text{5}\times \text{4}}$, and ${\left[\overset{\text{3}}{\underset{ij}{c}}\right]}_{\text{5}\times \text{4}}$ such that for all$\;i,j\in I_{\text{4}}$ and $k\in I_{\text{3}}$, $\overset{k}{\underset{\text{0}\text{1}}{c}}≺\overset{k}{\underset{\text{0}\text{2}}{c}}≺\overset{k}{\underset{\text{0}\text{3}}{c}}≺\overset{k}{\underset{\text{0}\text{4}}{c}}$, $\overset{k}{\underset{ii}{c}}=\lambda \in [\text{0},\text{1}]$, and if i≠ j, then $\;\overset{k}{\underset{ij}{c}}=\text{0}$. Therefore, $\overset{k}{\underset{\text{0}\text{1}}{c}}\overset{k}{\underset{\text{1}\text{1}}{c}}≺\overset{k}{\underset{\text{0}\text{2}}{c}}\overset{k}{\underset{\text{2}\text{2}}{c}}≺\overset{k}{\underset{\text{0}\text{3}}{c}}\overset{k}{\underset{\text{3}\text{3}}{c}}≺\overset{k}{\underset{\text{0}\text{4}}{c}}\overset{k}{\underset{\text{4}\text{4}}{c}}$ and if i≠ j, then $\;\overset{k}{\underset{\text{0}j}{c}}\overset{k}{\underset{ij}{c}}=\text{0}$, for all $\;j\in I_{\text{4}}$ and $k\in I_{\text{3}}$. For each *fpfs*-matrix herein, the ranking order of alternatives is $u_{\text{1}}≺u_{\text{2}}≺u_{\text{3}}≺u_{\text{4}}$. For example,


$$\begin{array}{c} \left[\overset{\text{1}}{\underset{ij}{c}}\right]:=[\begin{array}{cccc} \text{0}.\text{6}\text{0} & \text{0}.\text{7}\text{0} & \text{0}.\text{8}\text{0} & \text{0}.\text{9}\text{0}\\ \text{1} & \text{0} & \text{0} & \text{0}\\ \text{0} & \text{1} & \text{0} & \text{0}\\ \text{0} & \text{0} & \text{1} & \text{0}\\ \text{0} & \text{0} & \text{0} & \text{1} \end{array}],\;\left[\overset{\text{2}}{\underset{ij}{c}}\right]:=[\begin{array}{cccc} \text{0}.\text{4}\text{0} & \text{0}.\text{5}\text{0} & \text{0}.\text{6}\text{0} & \text{0}.\text{7}\text{0}\\ \text{1} & \text{0} & \text{0} & \text{0}\\ \text{0} & \text{1} & \text{0} & \text{0}\\ \text{0} & \text{0} & \text{1} & \text{0}\\ \text{0} & \text{0} & \text{0} & \text{1} \end{array}],\text{ and }\left[\overset{\text{3}}{\underset{ij}{c}}\right]:=[\begin{array}{cccc} \text{0}.\text{2}\text{0} & \text{0}.\text{3}\text{0} & \text{0}.\text{4}\text{0} & \text{0}.\text{5}\text{0}\\ \text{1} & \text{0} & \text{0} & \text{0}\\ \text{0} & \text{1} & \text{0} & \text{0}\\ \text{0} & \text{0} & \text{1} & \text{0}\\ \text{0} & \text{0} & \text{0} & \text{1} \end{array}] \end{array}$$

### 3.4. Test Case 4

Test Case 4 constructs three *fpfs*-matrices ${\left[\overset{\text{1}}{\underset{ij}{d}}\right]}_{\text{5}\times \text{4}}$, ${\left[\overset{\text{2}}{\underset{ij}{d}}\right]}_{\text{5}\times \text{4}}$, and ${\left[\overset{\text{3}}{\underset{ij}{d}}\right]}_{\text{5}\times \text{4}}$ such that for all$\;i,j\in I_{\text{4}}$ and $k\in I_{\text{3}}$, $\overset{k}{\underset{\text{0}\text{4}}{d}}≺\overset{k}{\underset{\text{0}\text{3}}{d}}≺\overset{k}{\underset{\text{0}\text{2}}{d}}≺\overset{k}{\underset{\text{0}\text{1}}{d}}$, $\overset{k}{\underset{ii}{d}}=\lambda \in [\text{0},\text{1}]$, and if i≠ j, then $\overset{k}{\underset{ij}{d}}=\text{0}$. Therefore, $\overset{k}{\underset{\text{0}\text{4}}{d}}\overset{k}{\underset{\text{4}\text{4}}{d}}≺\overset{k}{\underset{\text{0}\text{3}}{d}}\overset{k}{\underset{\text{3}\text{3}}{d}}≺\overset{k}{\underset{\text{0}\text{3}}{d}}\overset{k}{\underset{\text{3}\text{3}}{d}}≺\overset{k}{\underset{\text{0}\text{1}}{d}}\overset{k}{\underset{\text{1}\text{1}}{d}}$ and if i≠ j, then $\overset{k}{\underset{\text{0}j}{d}}\overset{k}{\underset{ij}{d}}=\text{0}$, for all$\;j\in I_{\text{4}}$ and $k\in I_{\text{3}}$. For each *fpfs*-matrix herein, the ranking order of alternatives is $u_{\text{4}}≺u_{\text{3}}≺u_{\text{2}}≺u_{\text{1}}$. For example,


$$\begin{array}{c} \left[\overset{\text{1}}{\underset{ij}{d}}\right]:=[\begin{array}{cccc} \text{0}.\text{9}\text{0} & \text{0}.\text{8}\text{0} & \text{0}.\text{7}\text{0} & \text{0}.\text{6}\text{0}\\ \text{1} & \text{0} & \text{0} & \text{0}\\ \text{0} & \text{1} & \text{0} & \text{0}\\ \text{0} & \text{0} & \text{1} & \text{0}\\ \text{0} & \text{0} & \text{0} & \text{1} \end{array}],\;\left[\overset{\text{2}}{\underset{ij}{d}}\right]:=[\begin{array}{cccc} \text{0}.\text{7}\text{0} & \text{0}.\text{6}\text{0} & \text{0}.\text{5}\text{0} & \text{0}.\text{4}\text{0}\\ \text{1} & \text{0} & \text{0} & \text{0}\\ \text{0} & \text{1} & \text{0} & \text{0}\\ \text{0} & \text{0} & \text{1} & \text{0}\\ \text{0} & \text{0} & \text{0} & \text{1} \end{array}],\text{ and }\left[\overset{\text{3}}{\underset{ij}{d}}\right]:=[\begin{array}{cccc} \text{0}.\text{5}\text{0} & \text{0}.\text{4}\text{0} & \text{0}.\text{3}\text{0} & \text{0}.\text{2}\text{0}\\ \text{1} & \text{0} & \text{0} & \text{0}\\ \text{0} & \text{1} & \text{0} & \text{0}\\ \text{0} & \text{0} & \text{1} & \text{0}\\ \text{0} & \text{0} & \text{0} & \text{1} \end{array}] \end{array}$$

### 3.5. Test Case 5

Test Case 5 constructs three *fpfs*-matrices ${\left[\overset{\text{1}}{\underset{ij}{e}}\right]}_{\text{5}\times \text{4}}$, ${\left[\overset{\text{2}}{\underset{ij}{e}}\right]}_{\text{5}\times \text{4}}$, and ${\left[\overset{\text{3}}{\underset{ij}{e}}\right]}_{\text{5}\times \text{4}}$ such that for all$\;i,j\in I_{\text{4}}$ and $k\in I_{\text{3}}$, $\overset{k}{\underset{ij}{e}}=\lambda \in [\text{0},\text{1}]$. For each *fpfs*-matrix herein, the ranking order of alternatives is $u_{\text{1}}\approx u_{\text{2}}\approx u_{\text{3}}\approx u_{\text{4}}$. Here, ≈ denotes the same ranking order. For example,


$$\begin{array}{c} \left[\overset{\text{1}}{\underset{ij}{e}}\right]:=[\begin{array}{cccc} \text{0}.\text{5}\text{0} & \text{0}.\text{5}\text{0} & \text{0}.\text{5}\text{0} & \text{0}.\text{5}\text{0}\\ \text{0}.\text{5}\text{0} & \text{0}.\text{5}\text{0} & \text{0}.\text{5}\text{0} & \text{0}.\text{5}\text{0}\\ \text{0}.\text{5}\text{0} & \text{0}.\text{5}\text{0} & \text{0}.\text{5}\text{0} & \text{0}.\text{5}\text{0}\\ \text{0}.\text{5}\text{0} & \text{0}.\text{5}\text{0} & \text{0}.\text{5}\text{0} & \text{0}.\text{5}\text{0}\\ \text{0}.\text{5}\text{0} & \text{0}.\text{5}\text{0} & \text{0}.\text{5}\text{0} & \text{0}.\text{5}\text{0} \end{array}],\;\left[\overset{\text{2}}{\underset{ij}{e}}\right]:=[\begin{array}{cccc} \text{0}.\text{5}\text{0} & \text{0}.\text{5}\text{0} & \text{0}.\text{5}\text{0} & \text{0}.\text{5}\text{0}\\ \text{0}.\text{5}\text{0} & \text{0}.\text{5}\text{0} & \text{0}.\text{5}\text{0} & \text{0}.\text{5}\text{0}\\ \text{0}.\text{5}\text{0} & \text{0}.\text{5}\text{0} & \text{0}.\text{5}\text{0} & \text{0}.\text{5}\text{0}\\ \text{0}.\text{5}\text{0} & \text{0}.\text{5}\text{0} & \text{0}.\text{5}\text{0} & \text{0}.\text{5}\text{0}\\ \text{0}.\text{5}\text{0} & \text{0}.\text{5}\text{0} & \text{0}.\text{5}\text{0} & \text{0}.\text{5}\text{0} \end{array}],\text{ and }\left[\overset{\text{3}}{\underset{ij}{e}}\right]=[\begin{array}{cccc} \text{0}.\text{5}\text{0} & \text{0}.\text{5}\text{0} & \text{0}.\text{5}\text{0} & \text{0}.\text{5}\text{0}\\ \text{0}.\text{5}\text{0} & \text{0}.\text{5}\text{0} & \text{0}.\text{5}\text{0} & \text{0}.\text{5}\text{0}\\ \text{0}.\text{5}\text{0} & \text{0}.\text{5}\text{0} & \text{0}.\text{5}\text{0} & \text{0}.\text{5}\text{0}\\ \text{0}.\text{5}\text{0} & \text{0}.\text{5}\text{0} & \text{0}.\text{5}\text{0} & \text{0}.\text{5}\text{0}\\ \text{0}.\text{5}\text{0} & \text{0}.\text{5}\text{0} & \text{0}.\text{5}\text{0} & \text{0}.\text{5}\text{0} \end{array}] \end{array}$$

According to Table 2, 35 algorithms passed all test cases. Among 35 SDM algorithms, 12 are based on single *fpfs*-matrices, nine are based on double *fpfs*-matrices, and 14 are based on multiple *fpfs*-matrices. Parameters are predetermined and used as α=0.5, β=0.5, κ=0.4, λ=0.5, $\lambda_{\text{1}}=[\text{1}\;\text{1}\;\text{1}\;\text{1}]$, $\lambda_{\text{2}}=[\text{0}.\text{2}\text{5}\;\text{0}.\text{2}\text{5}\;\text{0}.\text{2}\text{5}\;\text{0}.\text{2}\text{5}]$, $\lambda_{\text{3}}=[\text{0}.\text{5}\;\text{0}.\text{5}\;\text{0}.\text{5}\;\text{0}.\text{5}]$, q=2, R={1, 2, 3, 4}, and w=[0.34 0.34 0.34].

**Table 2:** SDM methods based on single, double, and multiple *fpfs*-matrices, which pass five tests.

	Algorithms\Test Cases	Single *fpfs-*Matrices	Double *fpfs-*Matrices	Multiple *fpfs-*Matrices	Test Case 1	Test Case 2	Test Case 3	Test Case 4	Test Case 5	Passed Test Numbers
1	CXL13(λ)	✓			✓	✓	✓	✓	✓	5
2	WQ14(κ)	✓			✓	✓	✓	✓	✓	5
3	YHX14(α,β)	✓			✓	✓	✓	✓	✓	5
4	DC15(α)	✓			✓	✓	✓	✓	✓	5
5	ZZ19(λ,γ)	✓			✓	✓	✓	✓	✓	5
6	CEC11, [AC16, AC16/2, RH16]	✓			✓	✓	✓	✓	✓	5
7	MBR01, [RK16]	✓			✓	✓	✓	✓	✓	5
8	G17(R)	✓			✓	✓	✓	✓	✓	5
9	LQP17(w)	✓			✓	✓	✓	✓	✓	5
10	[TOD17] (NRM16(𝐼𝑛)),KM11(𝐼𝑛))	✓			✓	✓	✓	✓	✓	5
11	LL18(𝝀)	✓			✓	✓	✓	✓	✓	5
12	A19(R,w,λ1,λ2,λ3,λ4,λ5)	✓			✓	✓	✓	✓	✓	5
13	CXL13/2(λ)		✓		✓	✓	✓	✓	✓	5
14	HG13		✓		✓	✓	✓	✓	✓	5
15	[GDC14, RH16/2,] and MRB02		✓		✓	✓	✓	✓	✓	5
16	ZXZ15(α)		✓		✓	✓	✓	✓	✓	5
17	VMH16		✓		✓	✓	✓	✓	✓	5
18	RH17		✓		✓	✓	✓	✓	✓	5
19	AKO18a		✓		✓	✓	✓	✓	✓	5
20	AKO18o		✓		✓	✓	✓	✓	✓	5
21	RH18		✓		✓	✓	✓	✓	✓	5
22	SM13(w,α)			✓	✓	✓	✓	✓	✓	5
23	NKY17 (λ), [GDC14/2(λ)]			✓	✓	✓	✓	✓	✓	5
24	Z14/2			✓	✓	✓	✓	✓	✓	5
25	MR13, [NB14], [AM16]			✓	✓	✓	✓	✓	✓	5
26	MR13/2,[AM16/2]			✓	✓	✓	✓	✓	✓	5
27	RS16			✓	✓	✓	✓	✓	✓	5
28	AT18(λ)			✓	✓	✓	✓	✓	✓	5
29	P18			✓	✓	✓	✓	✓	✓	5
30	A19/2(R)			✓	✓	✓	✓	✓	✓	5
31	SS19/2			✓	✓	✓	✓	✓	✓	5
32	SS19/3			✓	✓	✓	✓	✓	✓	5
33	SS19/4			✓	✓	✓	✓	✓	✓	5
34	S19/5(w).			✓	✓	✓	✓	✓	✓	5
35	ZCW19(δ,θ)			✓	✓	✓	✓	✓	✓	5
	Total	12	9	14						35

## 4. Classification of Configured SDM Methods

The study [64], which classifies classical MCDM methods into value/benefit-based, simple problem-solving, superiority-based problem-solving, and interactive problem-solving, inspired us in classifying SDM methods. It is crucial to classify SDM methods using soft set theory, a newly explored area, based on problem type and structure, thereby enabling the classification of SDM algorithms. During the classification, we considered the empirical study in which it was first used, the mathematical operator utilized therein, its performance in the defined test scenarios, the decision-making model, and the parameters employed. Based on the *fpfs*-matrices, 74 algorithms were configured and tabulated by their matrix structures, as shown in the tables. It discusses the original concept in depth, including the *fpfs*-based matrices, the decision model, and the mathematical operations herein.

We classified the methods based on the following criteria:

***Empirical study:*** Each method and its corresponding application areas have been examined.

***Operation:*** It denotes the mathematical operation used by that algorithm in the background.

***Number of tests passed:*** Five test scenarios have been defined to eliminate unsuccessful algorithms. These scenarios are designed so that an expert can naturally list alternatives that serve as the basis for test cases. The criteria specify the number of tests completed, thereby facilitating evaluation.

***Originality of Concept:*** Some SDM algorithms based on soft sets, fuzzy soft sets, fuzzy parameterized soft sets, *fpfs*-sets, soft matrices, and fuzzy soft matrices have been implemented using *fpfs*-matrices.

***Decision Model:*** The mathematics used by algorithms indicates the decision model.

***Parameter:*** It denotes parameter structures used in the algorithm.

Tables 3–5 detail SDM methods, including their abbreviations, the concepts they are based on, the decision model and mathematical operators they employ, the first empirical study in which they were used, and their parameters.

**Table 3:** Classification of the SDM methods utilizing single *fpfs*-matrices.

Algorithms	Originality of Concept	Decision Model	Operation	Empirical Study	Parameter
CXL13(λ)	fuzzy soft set	spatial distance to an optimal solution	distance-based aggregation	none	optimum solution matrix λ
WQ14(κ)	fuzzy soft sets	belief function-based clustering	belief function	investment decision	Bias coefficient chosen by the decision-maker κ in [0,1]
XWL14(α, q), LWX15(α, q), T15(α, q)	fuzzy soft sets	Grey relational analysis with the Dempster-Shafer theory of evidence	Grey relational analysis and belief function	medical diagnosis	α in real in [0,1], q in a positive natural number
YHX14(α, β)	fuzzy soft sets	Grey relational analysis	Grey relational analysis	quality selection of wine	α and β and in real in [0,1]
A15	fuzzy soft sets	sum of rows	comparison matrix, row sum, and dominant eigenvalues	none	–
DC15(α)	fuzzy parameterized soft sets	aggregation	AND-product and weighted aggregation	car selection	–
XHL15(α, q)	fuzzy soft matrices	Grey relational analysis	Grey relational analysis	medical diagnosis	α in real in [0,1] q in a positive natural number
ZZ19(λ, γ)	fuzzy soft sets	Min-Max and Max-Min	β-covering sets	recruitment problem	λ in real in (0,1) and Weight matrix γ in [0,1]
CEC11, AC16, AC16/2, RH16	soft sets and soft matrices	aggregation	aggregation	recruitment problem	–
NRM16(R)	fuzzy soft sets	aggregation	weighted product aggregation	Selecting an investment area	Indices Sets R
MBR01, RK16	fuzzy soft sets	binary comparison of the alternatives	comparison matrix	Selecting a social network	–
WHXDD16	soft matrices	aggregation with Dempster-Shafer theory	belief function	medical diagnosis	–
G17(R)	soft sets	aggregation	indices set-based weighted aggregation	house selection	Index sets R
LQP17(w)	fuzzy soft sets	aggregation	distance-based aggregation	none	Optimum solution matrix w
KM11($I_{n}$)	fuzzy soft sets	aggregation	indices set-based weighted-product aggregation	Stock management	–
KT18(R), MS10(R)	fuzzy soft sets	aggregation	indices set-based weighted aggregation	medical diagnosis	Index sets R
KT18/2(R) KM11	fuzzy soft sets	aggregation	indices set-based weighted-product aggregation	life ınsurances	–
LL18(λ)	fuzzy soft sets	TOPSIS	TOPSIS with improved entropy weight.	None	λ in real in (0,1)
X18	hybrid fuzzy set	aggregation	Dempster’s rule, Deng entropy, fuzzy preference relations	medical diagnosis	–
A19($R,w,\lambda_{\text{1}},\lambda_{\text{2}},\lambda_{\text{3}},\lambda_{\text{4}},\lambda_{\text{5}}$)	soft sets	aggregation	indices set-based weighted aggregation	house selection	Weight matrix w in [0,1] and $\lambda_{\text{1}},\lambda_{\text{2}},\lambda_{\text{3}},\lambda_{\text{4}},\lambda_{\text{5}}\in R$, Index sets R
MLQFG19, FJLL10/2m	fuzzy soft sets	aggregation	weighted aggregation	similarity of the websites	–

**Table 4:** Classification of the SDM methods utilizing double fpfs-matrices.

Algorithms	Originality of Concept	Decision Model	Operation	Empirical Study	Parameter
BSD13, SR15, NS11	fuzzy soft matrices	aggregation	complement union and weighted aggregation	health environment	–
CXL13/2(λ)	fuzzy soft sets	spatial distance to an optimal solution	mean and distance-based aggregation.	none	Optimum solution matrix λ
HG13	soft sets	equivalence relation	weighted sum and relation	pruning method scheme	–
GDC14, RH16/2, MRB02	soft sets and fuzzy soft matrices	aggregation	weighted aggregation	car selection	Optimum solution matrix λ
K14	soft sets	aggregation	weighted aggregation	house selection	–
MM14, CCE10	fuzzy parameterized soft sets and *fpfs-*sets	aggregation	weighted aggregation	Financial decision-making problems	–
XWL14/2(α,q), LWX15/2(α,q), T15/2(α,q)	fuzzy soft sets	Grey relational analysis with the Dempster-Shafer Theory	Grey relational analysis and belief function	medical diagnosis	α in real in [0,1] q in a positive natural number
HJ15(λ), H16(λ), CE10a	fuzzy soft sets	Uni-Int	AND-product	supplier selection	Weight matrix (λ) in [0,1]
XHL15/2(α,q)	fuzzy soft matrices	Grey relational analysis	AND-product and grey relational Analysis	medical diagnosis	α in real in [0,1] q in a positive natural number
ZXZ15(α)	*fpfs-*sets	aggregation	weighted aggregation (AND-Mean and OR-Mean Decision Making)	position selection	α in real in [0,1]
ZXZ15/2(α)	*fpfs*-sets	aggregation	weighted aggregation (AND-Mean and OR-Mean Decision Making)	position selection	α in real in [0,1]
ZZ15/2(λ)	fuzzy soft sets and rough soft sets	aggregation	weighted aggregation	material selection	Weight matrix λ
RH16/3(R)	*fpfs* Sets	binary comparison of the Alternatives	comparison matrix	car selection	Index sets R
VMH16	fuzzy soft sets	Max-Max	mean product	job candidate (group decision)	–
WHXDD16/2	soft matrices	aggregation with Dempster-Shafer Theory	belief function and AND-product	medical diagnosis.	–
RH17	*fpfs-*sets	aggregation	weighted aggregation	student selection	–
AKO18a	soft matrices	aggregation	AND product and AND-NOT product	country selection	–
AKO18o	soft matrices	aggregation	OR-product and OR-NOT product	country selection	–
RH18	fuzzy soft sets	aggregation	weighted aggregation	none	–
X18/2	hybrid fuzzy soft sets	aggregation	Dempster’s rule, Deng entropy, fuzzy preference relations, and AND-product	medical diagnosis	–

**Table 5:** SDM methods utilizing multiple *fpfs*-matrices.

Algorithms	Originality of Concept	Decision Model	Operation	Empirical Study	Parameter
GLF13(R)	fuzzy soft sets	order relations	Soft information order	house selection	Index sets R
SM13(w,α)	fuzzy soft sets	a set-theoretic similarity measure	similarity and collective coefficient	The company’s staff selection	Weight matrix w and real number α in [0,1]
GDC14/2(λ) NKY17(λ)	fuzzy soft sets	binary comparison of alternatives	comparison matrix	car selection and laptop selection	Weight matrix λ
Z14/2	soft sets	aggregation	weighted aggregation	recruitment problem	–
A16	fuzzy soft sets	binary comparison of the alternatives	comparison matrix	none	–
MR13, NB14, AM16	fuzzy soft matrices	aggregation	weighted aggregation	selecting water purifiers, candidate selection	–
MR13/2, AM16/2	fuzzy soft matrices	aggregation	weighted aggregation	selecting water purifiers, candidate selection	–
MR13/3, AM16/3	fuzzy soft matrices	aggregation	weighted aggregation	selecting water purifiers, candidate selection	–
RS16	fuzzy soft matrices	aggregation	weighted aggregation	–	–
SMT16	*fpfs*-sets	binary comparison of the alternatives	comparison matrix	job candidate (group decision)	–
AM17(∅,∅,∅)	fuzzy soft sets	aggregation	arithmetic mean	car selection	Index sets R
AM17/2(∅,∅,∅)	fuzzy soft sets	aggregation	geometric mean	car selection	Index sets R
AM17/3(5,∅,∅,∅)	fuzzy soft sets	aggregation	Zadeh’s Fuzzy complements	car selection	Index sets R and λ∈(−1,∞)
AM17/4(5,∅,∅,∅)	fuzzy soft sets	aggregation	Sugeno class of fuzzy complements	car selection	Index sets R and λ∈(−1,∞)
AM17/5 (5,∅,∅,∅)	fuzzy soft sets	aggregation	Yager’s class of fuzzy complements	car selection	Indices sets R and λ∈(−1,∞)
AM17/6 (5,∅,∅,∅)	fuzzy soft sets	aggregation	Yager’s class of fuzzy complements	car selection	Indices sets R and λ∈(−1,∞)
EC17(λ)	fuzzy soft sets	TOPSIS and grey relational analysis	TOPSIS and grey relational analysis	drug selection	λ ∈ (0,1)
AT18(λ)	fuzzy soft sets	aggregation	weighted aggregation	portfolio selection	λ∈(0,1)
P18, CEC11	fuzzy soft sets	aggregation	aggregation	city selection (office)	–
PS18	fuzzy soft sets	aggregation	aggregation	player selection	–
RHF18	*fpfs-sets*	aggregation	aggregation support set	none	–
A19/2(R)	fuzzy soft sets	aggregation	Indices Set-based weighted product aggregation	house selection	Index sets R
SS19	fuzzy soft sets	binary comparison of the alternatives	comparison matrix	job candidate	–
SS19/2	fuzzy soft sets	aggregation	weighted aggregation	performance evaluation	–
SS19/3	fuzzy soft sets	aggregation	weighted aggregation	performance evaluation	–
SS19/4	fuzzy soft sets	aggregation	weighted aggregation	performance evaluation	–
S19/5(w).	fuzzy soft sets	aggregation	weighted aggregation	ranking of public health centers	Weight matrix w in [0,1]
WQ19	fuzzy soft sets	Max-Min	modal-style Operators	none	–
ZCW19(δ,θ)	fuzzy soft Sets	aggregation	weighted aggregation	none	Weight matrix (δ,θ) in [0,1]
MD18, ND18	fuzzy soft Sets	aggregation	weighted aggregation	none	–
KAS18aa	*fpfs*-matrices	Max-Min	AND- product Fs-Matrices	holiday selection	–
KAS18aa/2	*fpfs*-matrices	Max-Min	AND- product Fs-matrices	holiday selection	–

## 5. Performance Comparison of SDM Methods Using Machine Learning

Table 6 presents the properties of the datasets used in the simulation herein: Parkinsons[sic], Wine, Sonar, Ecoli, Hayes-Roth, Libras Movement, Teaching, Ionosphere, Whosaler, and Glass. We subsequently present the mathematical notation for the performance metrics to compare the aforesaid methods. To ensure consistency and fair benchmarking, all ten UCI datasets underwent a standardized preprocessing step. This included feature fuzzification according to Definition 3 and, when needed, normalization as outlined in Definition 4, converting raw attributes into compatible *fpfs*-matrix structures before classification.

**Table 6:** Description of UCI data sets.

No	Dataset	# Instances	# Attributes	# Class	Balanced/Imbalanced	References
1	Parkinson[sic]	195	22	2	Imbalanced	Little (2007) [65]
2	Wine	178	13	3	Imbalanced	Aeberhard & Forina (1992) [66]
3	Sonar	208	60	2	Imbalanced	Sejnowski & Gorman (1988) [67]
4	Ecoli	336	7	7	Imbalanced	Nakai (1996) [68]
5	Hayes Roth	132	5	3	Imbalanced	Hayes & Hayes (1977) [69]
6	Libras Movement	360	90	15	Balanced	Dias et al. (2009) [70]
7	Teaching	151	5	3	Imbalanced	Loh (1997) [71]
8	Ionosphere	351	34	2	Imbalanced	Sigillito et al. (1989) [72]
9	Whosaler	440	7	3	Imbalanced	Cardoso (2013) [73]
10	Glass	214	9	7	Imbalanced	German (1987) [74]

# stands for “number of.”

The FPFS-CMC algorithm is presented in Algorithm 1. It uses the Pearson correlation coefficient to estimate feature weights based on the impact of the parameters on classification. Then, it creates two *fpfs*-matrices, one for training and one for testing, by fuzzifying the features of the training and testing samples based on their weights. It then constructs a comparison matrix by calculating pseudo-similarities between the training and testing *fpfs*-matrices. Afterward, the standard deviation is calculated for each column of the comparison matrix to obtain parameter weights. The comparison *fpfs-*matrix is then constructed by hybridizing the parameter weights and the comparison matrix. After that, the sMBR01 algorithm is applied to the comparison *fpfs*-matrix to obtain the optimal training sample. Then, the class label of the optimal training sample is assigned to the testing sample. This process is repeated for all the testing samples.

### Algorithm 1. FPFS-CMC Algorithm’s Steps [32]

$Input:\;{\left(D_{train}\;\right)}_{m_{\text{1}}xn}\;,C_{m\text{1}\times \text{1}}\;,{\left(D_{test}\right)}_{m\text{1}\times \text{1}}$

$Output:\;\overset{\prime }{\underset{m_{\text{2}}x\text{1}}{T}}$

**Procedure** FPFC-CMC($D_{train},C,D_{test})$

1. Compute  fw using $\;D_{train}\;$ and  C

2. Compute feature fuzzification of $\;D_{train}\;$ and $\;D_{test}\;,$ namely $\;{\tilde{D}}_{train}\;$ and $\;{\tilde{D}}_{test}\;$

3. **for**  i from  1 to $\;m_{\text{2}}\;$ do

4.  Compute the testing *fpfs*-matrix $\;\left[a_{ij}\right]\;$using  fw and $\;{\tilde{D}}_{i-test}\;$

5.  **for**  j  from  1 to $\;m_{\text{1}}\;$ do

6.  Compute the training *fpfs*-matrix $\;\left[b_{ij}\right]\;$using  fw and ${\tilde{D}}_{j-train}\;$

7.  $f_{j\text{1}}\;\leftarrow \;s_{H}([a_{ij}],[b_{ij}])$

8.  $\;f_{j\text{2}}\;\leftarrow \;s_{C}([a_{ij}],[b_{ij}])$

9.  $\;f_{j\text{3}}\;\leftarrow \;s_{E}([a_{ij}],[b_{ij}])$

10. $f_{j\text{4}}\;\leftarrow \;s_{HS}([a_{ij}],[b_{ij}])$

11. $f_{j\text{5}}\;\leftarrow \;\overset{\text{3}}{\underset{M}{s}}([a_{ij}],[b_{ij}])$

**12. end for**

**13. for**  j from  1 to  5 do

$\begin{array}{c} \text{1}\text{4}.\;\;sd_{j}\;\leftarrow \;std(F^{j})\; \end{array}$

**15. end for**

**16.** $\begin{array}{c} pw\;\leftarrow \;\text{1}\;-\;\hat{sd}\;/\;\text{4}\; \end{array}$

17. Compute comparison *fpfs*-matrix $\;[g_{ij}]\;$ using pw and F

18. $\left[sk_{\text{1}},dm_{k\text{1}},op_{k\text{1}}]\;\leftarrow \text{ sMBR01}([g_{ij}]\right)$

19. $\overset{\prime }{\underset{i\text{1}}{t}}\;\leftarrow \;C(op_{\text{1}\text{1}},\text{1})$

**20. end for**

**21. return** $\;\overset{\prime }{\underset{m\text{2}\times \text{1}}{T}}$

**end procedure**

Commonly used performance metrics [75], such as accuracy (Acc) (Eq. 1 and 10), precision (Pre) (Eq. 2 and 11), recall (Rec) (Eq. 3 and 12), specificity (Spe) (Eq. 4 and 13) and F1-score (F1) (Eq. 5 and 14) are presented to compare the performance of the algorithms. The mathematical notations of these performance metrics are as follows:

n samples to be classified $X=\left\{x_{\text{1}},x_{\text{2}},\ldots ,x_{n}\right\}$ let it be denoted as. $Y=\left\{Y_{\text{1}},Y_{\text{2}},\ldots ,Y_{n}\right\}$ the correct classes of these instances, $\hat{Y}=\left\{{\hat{Y}}_{\text{1}},{\hat{Y}}_{\text{2}},\ldots ,{\hat{Y}}_{n}\right\}$ let i=1,….,l denote the predicted classes of these samples and l the total number of classes.

Considering datasets containing binary classes, the true positive (TP), true negative (TN), false positive (FP), and false negative (FN) values:


$$\begin{array}{c} \text{Acc}(Y,\hat{Y}):=\frac{TP+TN}{TP+TN+FP+FN} \end{array}$$ (1)

$$\begin{array}{c} \text{Pre}(Y,\hat{Y}):=\frac{TP}{TP+FP} \end{array}$$ (2)

$$\begin{array}{c} \text{Rec}(Y,\hat{Y}):=\frac{TP}{TP+FN} \end{array}$$ (3)

$$\begin{array}{c} \text{Spe}(Y,\hat{Y}):=\frac{TN}{TN+FP} \end{array}$$ (4)

$$\begin{array}{c} \text{F1}-\text{Score}(Y,\hat{Y}):=\frac{\text{2}TP}{\text{2}TP+FP+FN} \end{array}$$ (5)

such that


$$\begin{array}{c} TP:=\left|\left\{x_{t}|\text{1}\in Y_{t}\wedge \text{1}\in {\hat{Y}}_{t},\text{1}\leq t\leq n\right\}\right| \end{array}$$ (6)

$$\begin{array}{c} TN:=\left|\left\{x_{t}|\text{0}\notin Y_{t}\wedge \text{0}\notin {\hat{Y}}_{t},\text{1}\leq t\leq n\right\}\right| \end{array}$$ (7)

$$\begin{array}{c} FP:=\left|\left\{x_{t}|\text{0}\notin Y_{t}\wedge \text{1}\in {\hat{Y}}_{t},\text{1}\leq t\leq n\right\}\right| \end{array}$$ (8)

$$\begin{array}{c} FN:=\left|\left\{x_{t}|\text{1}\in Y_{t}\wedge \text{0}\notin {\hat{Y}}_{t},\text{1}\leq t\leq n\right\}\right| \end{array}$$ (9)

Considering datasets with multiple classes, i. True positive for the class $\left(TP_{i}\right)$, true negative $\left(TN_{i}\right)$, false positive $\left(FP_{i}\right)$ and false negative $\left(FN_{i}\right)$ Values of:


$$\begin{array}{c} \text{Acc}(Y,\hat{Y}):=\frac{\text{1}}{l}\sum \overset{l}{\underset{i=\text{1}}{\mathrm{\backslash nolimits}}}\;\frac{TP_{i}+TN_{i}}{TP_{i}+TN_{i}+FP_{i}+FN_{i}} \end{array}$$ (10)

$$\begin{array}{c} \text{Pre}(Y,\hat{Y}):=\frac{\text{1}}{l}\sum \overset{l}{\underset{i=\text{1}}{\mathrm{\backslash nolimits}}}\;\frac{TP_{i}}{TP_{i}+FP_{i}} \end{array}$$ (11)

$$\begin{array}{c} \text{Rec}(Y,\hat{Y}):=\frac{\text{1}}{l}\sum \overset{l}{\underset{i=\text{1}}{\mathrm{\backslash nolimits}}}\;\frac{TP_{i}}{TP_{i}+FN_{i}} \end{array}$$ (12)

$$\begin{array}{c} \text{Spe}(Y,\hat{Y}):=\frac{\text{1}}{l}\sum \overset{l}{\underset{i=\text{1}}{\mathrm{\backslash nolimits}}}\;\frac{TN_{i}}{TN_{i}+FP_{i}} \end{array}$$ (13)

$$\begin{array}{c} \text{F1}-\text{score}(Y,\hat{Y}):=\frac{\text{1}}{l}\sum \overset{l}{\underset{i=\text{1}}{\mathrm{\backslash nolimits}}}\;\frac{\text{2}TP_{i}}{\text{2}TP_{i}+FP_{i}+FN_{i}} \end{array}$$ (14)

such that


$$\begin{array}{c} TP_{i}:=\left|\left\{x_{t}|i\in Y_{t}\wedge i\in {\hat{Y}}_{t},\text{1}\leq t\leq n\right\}\right| \end{array}$$ (15)

$$\begin{array}{c} TN_{i}:=\left|\left\{x_{t}|i\notin Y_{t}\wedge i\notin {\hat{Y}}_{t},\text{1}\leq t\leq n\right\}\right| \end{array}$$ (16)

$$\begin{array}{c} FP_{i}:=\left|\left\{x_{t}|i\notin Y_{t}\wedge i\in {\hat{Y}}_{t},\text{1}\leq t\leq n\right\}\right| \end{array}$$ (17)

$$\begin{array}{c} FN_{i}:=\left|\left\{x_{t}|i\in Y_{t}\wedge i\notin {\hat{Y}}_{t},\text{1}\leq t\leq n\right\}\right| \end{array}$$ (18)

### 5.1. Illustrative Example of Algorithm 1: FPFS-CMC

A data matrix $D\;=\;{\left[d_{ij}\right]}_{\text{1}\text{5}\times \text{6}}$ sampled from the “Teaching Assistant Evaluation” dataset is provided below to demonstrate the implementation of the proposed method. This subset contains 15 samples distributed across three distinct classes (l = 3) as indicated in the final column: class one consists of five samples, class two includes four samples, and class three contains six samples. In the initial iteration of the five-fold cross-validation process, the resulting training matrix ${\left(D_{train}\right)}_{\text{1}\text{2}\times \;\text{5}}$, the class vector $C_{\text{1}\text{2}\times \;\text{1}}$, and the test matrix ${\left(D_{test}\right)}_{\text{3}\times \;\text{5}}$ are established. Additionally, the ground truth labels for the test samples are represented by the vector $T_{\text{3}\times \;\text{1}}$ to facilitate the final performance evaluation. These matrices and vectors are obtained as follows:


$$\begin{array}{c} D=[\begin{array}{ccccc} \text{1} & \text{2}\text{3} & \text{3} & \text{1} & \text{1}\text{9}\\ \text{2} & \text{1}\text{5} & \text{3} & \text{1} & \text{1}\text{7}\\ \text{1} & \text{2}\text{3} & \text{3} & \text{2} & \text{4}\text{9}\\ \text{1} & \text{5} & \text{2} & \text{2} & \text{3}\text{3}\\ \text{2} & \text{7} & \text{1}\text{1} & \text{2} & \text{5}\text{5}\\ \text{2} & \text{6} & \text{1}\text{7} & \text{2} & \text{4}\text{2}\\ \text{2} & \text{6} & \text{1}\text{7} & \text{2} & \text{4}\text{3}\\ \text{2} & \text{7} & \text{1}\text{1} & \text{2} & \text{1}\text{0}\\ \text{2} & \text{2}\text{2} & \text{3} & \text{2} & \text{4}\text{6}\\ \text{2} & \text{1}\text{3} & \text{3} & \text{1} & \text{1}\text{0}\\ \text{2} & \text{2}\text{1} & \text{2} & \text{2} & \text{4}\text{2}\\ \text{2} & \text{2}\text{2} & \text{3} & \text{2} & \text{2}\text{8}\\ \text{2} & \text{1}\text{1} & \text{1} & \text{2} & \text{5}\text{1}\\ \text{2} & \text{1}\text{8} & \text{5} & \text{2} & \text{1}\text{9}\\ \text{2} & \text{1}\text{3} & \text{1} & \text{2} & \text{3}\text{1} \end{array}],D_{train}=[\begin{array}{ccccc} \text{1} & \text{2}\text{3} & \text{3} & \text{1} & \text{1}\text{9}\\ \text{2} & \text{1}\text{5} & \text{3} & \text{1} & \text{1}\text{7}\\ \text{1} & \text{2}\text{3} & \text{3} & \text{2} & \text{4}\text{9}\\ \text{2} & \text{7} & \text{1}\text{1} & \text{2} & \text{5}\text{5}\\ \text{2} & \text{6} & \text{1}\text{7} & \text{2} & \text{4}\text{2}\\ \text{2} & \text{6} & \text{1}\text{7} & \text{2} & \text{4}\text{3}\\ \text{2} & \text{7} & \text{1}\text{1} & \text{2} & \text{1}\text{0}\\ \text{2} & \text{2}\text{2} & \text{3} & \text{2} & \text{4}\text{6}\\ \text{2} & \text{2}\text{1} & \text{2} & \text{2} & \text{4}\text{2}\\ \text{2} & \text{1}\text{1} & \text{1} & \text{2} & \text{5}\text{1}\\ \text{2} & \text{1}\text{8} & \text{5} & \text{2} & \text{1}\text{9}\\ \text{2} & \text{1}\text{3} & \text{1} & \text{2} & \text{3}\text{1} \end{array}],\;C=[\begin{array}{c} \text{3}\\ \text{3}\\ \text{3}\\ \text{3}\\ \text{2}\\ \text{2}\\ \text{2}\\ \text{2}\\ \text{1}\\ \text{1}\\ \text{1}\\ \text{1} \end{array}],D_{test}=[\begin{array}{ccccc} \text{1} & \text{5} & \text{2} & \text{2} & \text{3}\text{3}\\ \text{2} & \text{1}\text{3} & \text{3} & \text{1} & \text{1}\text{0}\\ \text{2} & \text{2}\text{2} & \text{3} & \text{2} & \text{2}\text{8} \end{array}],\;T=[\begin{array}{c} \text{3}\\ \text{2}\\ \text{1} \end{array}] \end{array}$$

As part of the procedural demonstration for the “Teaching Assistant Evaluation” dataset, the following refined text describes the specific stages of the FPFS-CMC algorithm: The matrices $D_{train}$, C, and $D_{test}$ are provided as inputs to the FPFS-CMC. Following the classification task, the ground truth class labels T are utilized to evaluate the performance of the integrated SDM methods. While several metrics, such as Acc, Pre, Rec, Spe, and F1-score, are commonly used for comprehensive performance analysis, this numerical example focuses specifically on Acc to demonstrate the method’s fundamental effectiveness.

Secondly, the feature weights (fw) are computed by analyzing the relationship between $D_{train}$ and C using the Pearson correlation coefficient. In the FPFS-CM, this correlation is utilized to quantify the statistical significance of each attribute relative to the class labels. The primary objective of obtaining these feature weights is to provide a weighted foundation for constructing the training and testing *fpfs*-matrices in the subsequent phases, ensuring that the decision-making process prioritizes the most influential features of the dataset.


$$\begin{array}{c} \left[{fw}_{\text{1}j}\right]=[\begin{array}{ccccc} \text{0}.\text{5}\text{4}\text{8} & \text{0}.\text{0}\text{7}\text{7} & \text{0}.\text{1}\text{9}\text{6} & \text{0}.\text{5}\text{4}\text{8} & \text{0}.\text{0}\text{2}\text{1} \end{array}] \end{array}$$

Thirdly, feature fuzzifications of $D_{train}$ and $D_{test}$ are computed as follows:


$$\begin{array}{c} {\tilde{D}}_{train}=[\begin{array}{ccccc} \text{0} & \text{1} & \text{0}.\text{1}\text{2}\text{5} & \text{0} & \text{0}.\text{2}\text{0}\text{0}\\ \text{1} & \text{0}.\text{5}\text{5}\text{6} & \text{0}.\text{1}\text{2}\text{5} & \text{0} & \text{0}.\text{1}\text{5}\text{6}\\ \text{0} & \text{1} & \text{0}.\text{1}\text{2}\text{5} & \text{1} & \text{0}.\text{8}\text{6}\text{7}\\ \text{1} & \text{0}.\text{1}\text{1}\text{1} & \text{0}.\text{6}\text{2}\text{5} & \text{1} & \text{1}\\ \text{1} & \text{0}.\text{0}\text{5}\text{6} & \text{1} & \text{1} & \text{0}.\text{7}\text{1}\text{1}\\ \text{1} & \text{0}.\text{0}\text{5}\text{6} & \text{1} & \text{1} & \text{0}.\text{7}\text{3}\text{3}\\ \text{1} & \text{0}.\text{1}\text{1}\text{1} & \text{0}.\text{6}\text{2}\text{5} & \text{1} & \text{0}\\ \text{1} & \text{0}.\text{9}\text{4}\text{4} & \text{0}.\text{1}\text{2}\text{5} & \text{1} & \text{0}.\text{8}\text{0}\text{0}\\ \text{1} & \text{0}.\text{8}\text{8}\text{9} & \text{0}.\text{6}\text{2}\text{5} & \text{1} & \text{0}.\text{7}\text{1}\text{1}\\ \text{1} & \text{0}.\text{3}\text{3}\text{3} & \text{0} & \text{1} & \text{0}.\text{9}\text{1}\text{1}\\ \text{1} & \text{0}.\text{7}\text{2}\text{2} & \text{0}.\text{2}\text{5}\text{0} & \text{1} & \text{0}.\text{2}\text{0}\text{0}\\ \text{1} & \text{0}.\text{4}\text{4}\text{4} & \text{0} & \text{1} & \text{0}.\text{4}\text{6}\text{7} \end{array}]\text{ and }{\tilde{D}}_{test}=[\begin{array}{ccccc} \text{0} & \text{0} & \text{0}.\text{0}\text{6}\text{3} & \text{1} & \text{0}.\text{5}\text{1}\text{1}\\ \text{1} & \text{0}.\text{4}\text{4}\text{4} & \text{0}.\text{1}\text{2}\text{5} & \text{0} & \text{0}\\ \text{1} & \text{0}.\text{9}\text{4}\text{4} & \text{0}.\text{1}\text{2}\text{5} & \text{1} & \text{0}.\text{4}\text{0}\text{0} \end{array}] \end{array}$$

In the subsequent phases, the training and testing samples from $D_{train}$ and $D_{test}$ undergo a feature fuzzification process to derive their fuzzy representations, denoted as ${\tilde{D}}_{train}$ and ${\tilde{D}}_{test}$, respectively. This transformation is a critical prerequisite for constructing the train and test *fpfs*-matrices. By mapping raw attribute values to a fuzzy membership space, the FPFS-CMC framework effectively captures inherent data uncertainties, providing a robust foundation for the multi-criteria classification process.

To illustrate the specific classification procedure, the following computational steps are executed for the first test sample (i=1):

Fourthly, for each training sample j, the test *fpfs*-matrix $\left[a_{ij}\right]$ and the corresponding training *fpfs*-matrix $\left[b_{ij}\right]$ are constructed utilizing the fuzzy representations ${\tilde{D}}_{i-test}$ and $D_{j-train}$, respectively. As a specific instance, for the first test sample (i=1) and the first training sample (j=1), the test *fpfs*-matrix $\left[a_{ij}\right]$ and the training *fpfs*-matrix $\left[b_{ij}\right]$ are generated as follows:


$$\begin{array}{c} \left[a_{ij}\right]=[\begin{array}{ccccc} \text{0}.\text{5}\text{4}\text{8} & \text{0}.\text{0}\text{7}\text{7} & \text{0}.\text{1}\text{9}\text{6} & \text{0}.\text{5}\text{4}\text{8} & \text{0}.\text{0}\text{2}\text{1}\\ \text{0} & \text{0} & \text{0}.\text{0}\text{6}\text{3} & \text{1} & \text{0}.\text{5}\text{1}\text{1} \end{array}] \end{array}$$

and


$$\begin{array}{c} \left[b_{ij}\right]=[\begin{array}{ccccc} \text{0}.\text{5}\text{4}\text{8} & \text{0}.\text{0}\text{7}\text{7} & \text{0}.\text{1}\text{9}\text{6} & \text{0}.\text{5}\text{4}\text{8} & \text{0}.\text{0}\text{2}\text{1}\\ \text{0} & \text{1} & \text{0}.\text{1}\text{2}\text{5} & \text{0} & \text{0}.\text{2}\text{0}\text{0} \end{array}] \end{array}$$

The first rows of the *fpfs*-matrices $\left[a_{ij}\right]$ and $\left[b_{ij}\right]$ consist of the $\left[{fw}_{ij}\right]$ calculated in the second step. The second rows of these matrices correspond to the fuzzy representations of the first test sample ${\tilde{D}}_{train}$ and the first training sample ${\tilde{D}}_{\text{1}-train}$, respectively.

In the fifth step, the matrix F is constructed for the $i^{th}$ test sample. Each row of this matrix represents the relationship between the test sample and a specific training sample j, while each column corresponds to one of the five distinct pseudo-similarity measures. For the first test sample (i=1), the $j^{th}$ row of the matrix F is computed by applying the following similarity functions between $\left[a_{ij}\right]$ and $\left[b_{ij}\right]$: Hamming pseudo-similarity, Chebyshev pseudo-similarity, Euclidean pseudo-similarity, Hausdorff pseudo-similarity, and Minkowski pseudo-similarity of *fpfs*-matrices. This systematic process continues for all training samples (j=1, 2, …, n), resulting in a comprehensive matrix F that captures the multifaceted proximity between the test instance and the entire training set.


$$\begin{array}{c} F=[\begin{array}{ccccc} \text{0}.\text{8}\text{7}\text{1} & \text{0}.\text{4}\text{5}\text{2} & \text{0}.\text{7}\text{5}\text{3} & \text{0}.\text{4}\text{5}\text{2} & \text{0}.\text{6}\text{7}\text{9}\\ \text{0}.\text{7}\text{6}\text{8} & \text{0}.\text{4}\text{5}\text{2} & \text{0}.\text{6}\text{5}\text{3} & \text{0}.\text{4}\text{5}\text{2} & \text{0}.\text{5}\text{9}\text{6}\\ \text{0}.\text{9}\text{8}\text{1} & \text{0}.\text{9}\text{2}\text{3} & \text{0}.\text{9}\text{6}\text{5} & \text{0}.\text{9}\text{2}\text{3} & \text{0}.\text{9}\text{5}\text{5}\\ \text{0}.\text{8}\text{6}\text{5} & \text{0}.\text{4}\text{5}\text{2} & \text{0}.\text{7}\text{5}\text{0} & \text{0}.\text{4}\text{5}\text{2} & \text{0}.\text{6}\text{7}\text{9}\\ \text{0}.\text{8}\text{5}\text{2} & \text{0}.\text{4}\text{5}\text{2} & \text{0}.\text{7}\text{4}\text{2} & \text{0}.\text{4}\text{5}\text{2} & \text{0}.\text{6}\text{7}\text{6}\\ \text{0}.\text{8}\text{5}\text{2} & \text{0}.\text{4}\text{5}\text{2} & \text{0}.\text{7}\text{4}\text{2} & \text{0}.\text{4}\text{5}\text{2} & \text{0}.\text{6}\text{7}\text{6}\\ \text{0}.\text{8}\text{6}\text{5} & \text{0}.\text{4}\text{5}\text{2} & \text{0}.\text{7}\text{5}\text{0} & \text{0}.\text{4}\text{5}\text{2} & \text{0}.\text{6}\text{7}\text{9}\\ \text{0}.\text{8}\text{7}\text{2} & \text{0}.\text{4}\text{5}\text{2} & \text{0}.\text{7}\text{5}\text{3} & \text{0}.\text{4}\text{5}\text{2} & \text{0}.\text{6}\text{8}\text{0}\\ \text{0}.\text{8}\text{7}\text{6} & \text{0}.\text{4}\text{5}\text{2} & \text{0}.\text{7}\text{5}\text{3} & \text{0}.\text{4}\text{5}\text{2} & \text{0}.\text{6}\text{8}\text{0}\\ \text{0}.\text{8}\text{8}\text{1} & \text{0}.\text{4}\text{5}\text{2} & \text{0}.\text{7}\text{5}\text{5} & \text{0}.\text{4}\text{5}\text{2} & \text{0}.\text{6}\text{8}\text{0}\\ \text{0}.\text{8}\text{7}\text{1} & \text{0}.\text{4}\text{5}\text{2} & \text{0}.\text{7}\text{5}\text{3} & \text{0}.\text{4}\text{5}\text{2} & \text{0}.\text{6}\text{8}\text{0}\\ \text{0}.\text{8}\text{8}\text{1} & \text{0}.\text{4}\text{5}\text{2} & \text{0}.\text{7}\text{5}\text{5} & \text{0}.\text{4}\text{5}\text{2} & \text{0}.\text{6}\text{8}\text{0} \end{array}] \end{array}$$

In the sixth step, a column-based standard deviation analysis is performed on the matrix F to determine the parameter weights (pw). For each of the five pseudo-similarity measures j=1, 2, …, 5),, the standard deviation ($sd_{j}$) is calculated to assess the dispersion of similarity scores across the training set.


$$\begin{array}{c} \left[{sd}_{\text{1}j}\right]=[\begin{array}{ccccc} \text{0}.\text{0}\text{4}\text{6} & \text{0}.\text{1}\text{3}\text{6} & \text{0}.\text{0}\text{7}\text{0} & \text{0}.\text{1}\text{3}\text{6} & \text{0}.\text{0}\text{8}\text{5} \end{array}] \end{array}$$

Sevently, these values are normalized to generate a parameter weight vector (pw), which quantifies the reliability and discriminative power of each similarity measure.


$$\begin{array}{c} \left[{pw}_{\text{1}j}\right]=[\begin{array}{ccccc} \text{1} & \text{0}.\text{7}\text{5}\text{0} & \text{0}.\text{9}\text{3}\text{3} & \text{0}.\text{7}\text{5}\text{0} & \text{0}.\text{8}\text{9}\text{1} \end{array}] \end{array}$$

In the eighth step, these weights are integrated with the matrix F to construct the comparison *fpfs*-matrix $\left[g_{ij}\right]$.


$$\left[g_{ij}]=[\begin{array}{ccccc} \text{1} & \text{0}.\text{7}\text{5}\text{0} & \text{0}.\text{9}\text{3}\text{3} & \text{0}.\text{7}\text{5}\text{0} & \text{0}.\text{8}\text{9}\text{1}\\ \text{0}.\text{8}\text{7}\text{1} & \text{0}.\text{4}\text{5}\text{2} & \text{0}.\text{7}\text{5}\text{3} & \text{0}.\text{4}\text{5}\text{2} & \text{0}.\text{6}\text{7}\text{9}\\ \text{0}.\text{7}\text{6}\text{8} & \text{0}.\text{4}\text{5}\text{2} & \text{0}.\text{6}\text{5}\text{3} & \text{0}.\text{4}\text{5}\text{2} & \text{0}.\text{5}\text{9}\text{6}\\ \text{0}.\text{9}\text{8}\text{1} & \text{0}.\text{9}\text{2}\text{3} & \text{0}.\text{9}\text{6}\text{5} & \text{0}.\text{9}\text{2}\text{3} & \text{0}.\text{9}\text{5}\text{5}\\ \text{0}.\text{8}\text{6}\text{5} & \text{0}.\text{4}\text{5}\text{2} & \text{0}.\text{7}\text{5}\text{0} & \text{0}.\text{4}\text{5}\text{2} & \text{0}.\text{6}\text{7}\text{9}\\ \text{0}.\text{8}\text{5}\text{2} & \text{0}.\text{4}\text{5}\text{2} & \text{0}.\text{7}\text{4}\text{2} & \text{0}.\text{4}\text{5}\text{2} & \text{0}.\text{6}\text{7}\text{6}\\ \text{0}.\text{8}\text{5}\text{2} & \text{0}.\text{4}\text{5}\text{2} & \text{0}.\text{7}\text{4}\text{2} & \text{0}.\text{4}\text{5}\text{2} & \text{0}.\text{6}\text{7}\text{6}\\ \text{0}.\text{8}\text{6}\text{5} & \text{0}.\text{4}\text{5}\text{2} & \text{0}.\text{7}\text{5}\text{0} & \text{0}.\text{4}\text{5}\text{2} & \text{0}.\text{6}\text{7}\text{9}\\ \text{0}.\text{8}\text{7}\text{2} & \text{0}.\text{4}\text{5}\text{2} & \text{0}.\text{7}\text{5}\text{3} & \text{0}.\text{4}\text{5}\text{2} & \text{0}.\text{6}\text{8}\text{0}\\ \text{0}.\text{8}\text{7}\text{6} & \text{0}.\text{4}\text{5}\text{2} & \text{0}.\text{7}\text{5}\text{3} & \text{0}.\text{4}\text{5}\text{2} & \text{0}.\text{6}\text{8}\text{0}\\ \text{0}.\text{8}\text{8}\text{1} & \text{0}.\text{4}\text{5}\text{2} & \text{0}.\text{7}\text{5}\text{5} & \text{0}.\text{4}\text{5}\text{2} & \text{0}.\text{6}\text{8}\text{0}\\ \text{0}.\text{8}\text{7}\text{1} & \text{0}.\text{4}\text{5}\text{2} & \text{0}.\text{7}\text{5}\text{3} & \text{0}.\text{4}\text{5}\text{2} & \text{0}.\text{6}\text{8}\text{0}\\ \text{0}.\text{8}\text{8}\text{1} & \text{0}.\text{4}\text{5}\text{2} & \text{0}.\text{7}\text{5}\text{5} & \text{0}.\text{4}\text{5}\text{2} & \text{0}.\text{6}\text{8}\text{0} \end{array}\right]$$

Finally, the comparison *fpfs*-matrix $\left[g_{ij}\right]$ is processed through the sMBR01 soft decision-making algorithm. The sMBR01 identifies the optimal training sample $\left(op_{k\text{1}}\right)$ by evaluating score functions within the matrix. For the $i^{th}$ test sample, sMBR01$\left([g_{ij}]\right)$ produces $\left[op_{\text{1}\text{1}}\right]=[\text{3}]$. Therefore, the class label of this selected training instance $c_{\text{6}\text{1}}=\text{3}$ is then assigned as the predicted label $\overset{\prime }{\underset{i\text{1}}{t}}$ for the $i^{th}$ test sample. This process is iteratively executed for all test instances until the final prediction vector $\overset{\prime }{\underset{\text{3}\times \;\text{1}}{T}}=[\begin{array}{c} \text{3}\\ \text{3}\\ \text{2} \end{array}]$ is generated, completing the classification cycle. Consequently, $\text{Acc}\left(T_{\text{3}\times \;\text{1}},\overset{\prime }{\underset{\text{3}\times \;\text{1}}{T}}\right)=\text{0}.\text{33}$ is obtained.

The performance comparisons of the aforesaid SDM methods on the datasets are presented in Tables 7–16.

**Table 7:** Performance comparisons of algorithms on the Parkinsons[sic] dataset.

Single *fpfs*-Matrices	Algorithms	Acc	Pre	Rec	Spe	F1-score	Ranking
CXL13(λ)	92.8205	84.5068	88.6667	94.1609	85.8321	11
WQ14(κ)	95.0769	88.6009	93.2000	95.6460	90.3367	5
YHX14(α,β)	95.2821	89.4874	92.8000	96.0460	90.6444	3
DC15(α)	93.4359	85.7650	89.9111	94.5655	87.1665	9
ZZ19(λ,γ)	93.1282	84.6013	89.9111	94.1609	86.5797	10
CEC11	95.1795	88.9039	93.2000	95.7839	90.5098	4
MBR01	94.4615	87.3397	92.3556	95.0989	89.1885	8
G17(R)	95.2821	89.2312	93.2000	95.9172	90.6993	2
LQP17(w)	95.2821	89.2312	93.2000	95.9172	90.6993	2
KM11(𝐼𝑛))	94.5641	87.8717	91.9556	95.3701	89.2883	7
LL18(𝝀)	94.8718	88.4675	92.4000	95.6414	89.8749	6
A19(R,w,λ1,λ2,λ3,λ4,λ5)	95.4872	90.3901	92.4000	96.4552	90.9574	1
**Double *fpfs*-Matrices**	CXL13/2(λ)	92.8205	84.5068	88.6667	94.1609	85.8321	4
	HG13	95.2821	89.2312	93.2000	95.9172	90.6993	1
	MRB02	95.2821	89.2312	93.2000	95.9172	90.6993	1
	ZXZ15(α)	95.2821	89.2312	93.2000	95.9172	90.6993	1
	VMH16	94.0513	87.9703	89.4667	95.5080	88.0329	3
	RH17	95.1795	88.9039	93.2000	95.7839	90.5098	2
	AKO18a	91.6923	81.7190	87.4222	93.0621	83.6980	5
	AKO18o	94.0513	87.9703	89.4667	95.5080	88.0329	3
	RH18	95.2821	89.2312	93.2000	95.9172	90.6993	1
**More than two *fpfs*-Matrices**	SM13(w,α)	94.7692	88.4312	91.9556	95.6460	89.6196	5
	NKY17(λ)	94.4615	87.3397	92.3556	95.0989	89.1885	6
	Z14/2	95.2821	89.2312	93.2000	95.9172	90.6993	1
	MR13	95.2821	89.2312	93.2000	95.9172	90.6993	1
	MR13/2	95.2821	89.2312	93.2000	95.9218	90.6813	2
	RS16	95.2821	89.2312	93.2000	95.9172	90.6993	1
	AT18(λ)	95.1795	88.9281	93.2000	95.7793	90.5261	3
	P18	95.1795	88.9039	93.2000	95.7839	90.5098	4
	A19/2(R)	94.0513	87.9703	89.4667	95.5080	88.0329	7
	SS19/2	95.1795	88.9281	93.2000	95.7793	90.5261	3
	SS19/3	95.0769	88.5281	93.2000	95.6414	90.3367	5
	SS19/4	95.0769	88.6009	93.2000	95.6460	90.3367	5
	S19/5(w).	95.2821	89.2312	93.2000	95.9172	90.6993	1
	ZCW19(δ,θ)	95.2821	89.2312	93.2000	95.9172	90.6993	1

**Table 8:** Performance comparisons of algorithms on the Wine dataset.

Single *fpfs*-Matrices	Algorithms	Acc	Pre	Rec	Spe	F1-score	Ranking
CXL13(λ)	96.8487	95.4678	95.9798	97.6773	95.2730	9
WQ14(κ)	97.6783	96.6501	97.0857	98.3146	96.5175	3
YHX14(α,β)	97.6042	96.5927	96.9582	98.2436	96.4063	4
DC15(α)	96.9228	95.6037	96.0909	97.7379	95.3841	8
ZZ19(λ,γ)	96.9228	95.6037	96.0909	97.7379	95.3841	8
CEC11	97.7545	96.7307	97.1746	98.3701	96.6317	2
MBR01	97.1556	95.9399	96.3867	97.9164	95.7333	7
G17(R)	97.5259	96.5041	96.9016	98.2010	96.2889	5
LQP17(w)	97.5259	96.5041	96.9016	98.2010	96.2889	5
KM11(𝐼𝑛))	97.4519	96.3828	96.8063	98.1498	96.1778	6
LL18(𝝀)	97.5259	96.4406	96.8952	98.2053	96.2889	5
A19(R,w,λ1,λ2,λ3,λ4,λ5)	98.0487	97.2201	97.5619	98.5710	97.0730	1
**Double *fpfs*-Matrices**	CXL13/2(λ)	96.8487	95.4678	95.9798	97.6773	95.2730	4
	HG13	97.5259	96.5041	96.9016	98.2010	96.2889	3
	MRB02	97.5259	96.5041	96.9016	98.2010	96.2889	3
	ZXZ15(α)	97.5259	96.5041	96.9016	98.2010	96.2889	3
	VMH16	98.0487	97.1822	97.5683	98.5734	97.0730	1
	RH17	97.7545	96.7307	97.1746	98.3701	96.6317	2
	AKO18a	96.5503	95.0321	95.4232	97.4382	94.8254	4
	AKO18o	98.0487	97.1822	97.5683	98.5734	97.0730	1
	RH18	97.5259	96.5041	96.9016	98.2010	96.2889	3
**More than two *fpfs*-Matrices**	SM13(w,α)	97.5259	96.4406	96.8952	98.2053	96.2889	4
	NKY17(λ)	97.1556	95.9399	96.3867	97.9164	95.7333	5
	Z14/2	97.5259	96.5041	96.9016	98.2010	96.2889	4
	MR13	97.5259	96.5041	96.9016	98.2010	96.2889	4
	MR13/2	97.6063	96.5519	96.9841	98.2590	96.4095	3
	RS16	97.5259	96.5041	96.9016	98.2010	96.2889	4
	AT18(λ)	97.5259	96.5041	96.9016	98.2010	96.2889	4
	P18	97.7545	96.7307	97.1746	98.3701	96.6317	2
	A19/2(R)	98.0487	97.1822	97.5683	98.5734	97.0730	1
	SS19/2	97.5259	96.5041	96.9016	98.2010	96.2889	4
	SS19/3	97.5259	96.5041	96.9016	98.2010	96.2889	4
	SS19/4	97.6063	96.5852	96.9841	98.2547	96.4095	3
	S19/5(w).	97.5259	96.5041	96.9016	98.2010	96.2889	4
	ZCW19(δ,θ)	97.5259	96.5041	96.9016	98.2010	96.2889	4

**Table 9:** Performance comparisons of algorithms on the Sonar dataset.

Single *fpfs*-Matrices	Algorithms	Acc	Pre	Rec	Spe	F1-score	Ranking
CXL13(λ)	83.2613	85.1346	78.1474	87.7708	81.1827	7
WQ14(κ)	85.7654	87.9004	81.2421	89.7708	84.0745	9
YHX14(α,β)	87.8862	91.5014	82.0947	92.9723	86.2911	1
DC15(α)	84.4088	86.6291	79.3684	88.8617	82.4380	11
ZZ19(λ,γ)	84.2184	86.4501	79.1684	88.6798	82.2238	5
CEC11	85.8630	87.9472	81.4526	89.7708	84.2201	6
MBR01	87.2149	89.2154	83.0947	90.8379	85.7545	2
G17(R)	85.8606	88.2285	81.0316	90.1265	84.1401	8
LQP17(w)	85.8606	88.2285	81.0316	90.1265	84.1401	8
KM11(𝐼𝑛))	85.5703	88.1247	80.4000	90.1344	83.7579	10
LL18(𝝀)	86.0511	88.2824	81.4421	90.1265	84.3859	4
A19(R,w,λ1,λ2,λ3,λ4,λ5)	87.2149	91.8559	80.0421	93.5020	85.3089	3
**Double *fpfs*-Matrices**	CXL13/2(λ)	83.2613	85.1346	78.1474	87.7708	81.1827	4
	HG13	85.8606	88.2285	81.0316	90.1265	84.1401	3
	MRB02	85.8606	88.2285	81.0316	90.1265	84.1401	3
	ZXZ15(α)	85.8606	88.2285	81.0316	90.1265	84.1401	3
	VMH16	86.9315	92.2436	79.0105	93.8656	84.8335	1
	RH17	85.8630	87.9472	81.4526	89.7708	84.2201	2
	AKO18a	82.5923	84.8560	76.7158	87.7628	80.3098	5
	AKO18o	86.9315	92.2436	79.0105	93.8656	84.8335	1
	RH18	85.8606	88.2285	81.0316	90.1265	84.1401	3
**More than two *fpfs*-Matrices**	SM13(w,α)	85.9535	88.0994	81.4421	89.9447	84.3019	5
	NKY17(λ)	87.2149	89.2154	83.0947	90.8379	85.7545	2
	Z14/2	85.8606	88.2285	81.0316	90.1265	84.1401	8
	MR13	85.8606	88.2285	81.0316	90.1265	84.1401	8
	MR13/2	86.1510	88.2898	81.6632	90.1265	84.5611	4
	RS16	85.8606	88.2285	81.0316	90.1265	84.1401	8
	AT18(λ)	85.9559	88.3843	81.0316	90.3083	84.2238	6
	P18	85.8630	87.9472	81.4526	89.7708	84.2201	7
	A19/2(R)	86.9315	92.2436	79.0105	93.8656	84.8335	3
	SS19/2	85.9559	88.3843	81.0316	90.3083	84.2238	6
	SS19/3	85.8583	88.3680	80.8211	90.3083	84.0964	9
	SS19/4	87.6911	89.9676	83.2947	91.5573	86.3145	1
	S19/5(w).	85.8606	88.2285	81.0316	90.1265	84.1401	8
	ZCW19(δ,θ)	85.8606	88.2285	81.0316	90.1265	84.1401	8

**Table 10:** Performance comparisons of algorithms on the Hayes dataset.

Single *fpfs*-Matrices	Algorithms	Acc	Pre	Rec	Spe	F1-score	Ranking
CXL13(λ)	84.9383	80.4004	76.4040	88.0352	77.4074	7
WQ14(κ)	84.9421	80.8087	76.5939	87.9869	77.4131	6
YHX14(α,β)	86.1576	83.2540	78.8929	88.8343	79.2365	1
DC15(α)	84.9383	80.4665	76.4040	88.0303	77.4074	7
ZZ19(λ,γ)	84.8395	80.5607	76.2707	87.9271	77.2593	8
CEC11	84.7331	80.4198	76.3152	87.8154	77.0997	9
MBR01	85.2384	80.8994	76.9818	88.2803	77.8575	2
G17(R)	85.0408	80.9198	76.7152	88.0654	77.5613	5
LQP17(w)	85.0408	80.9198	76.7152	88.0654	77.5613	5
KM11(𝐼𝑛))	85.1396	80.8776	77.0263	88.1605	77.7094	3
LL18(𝝀)	85.1320	81.4887	76.9253	88.1024	77.6980	4
A19(R,w,λ1,λ2,λ3,λ4,λ5)	86.1576	83.1379	78.8929	88.8392	79.2365	1
**Double *fpfs*-Matrices**	CXL13/2(λ)	84.9383	80.4004	76.4040	88.0352	77.4074	3
	HG13	85.0408	80.9198	76.7152	88.0654	77.5613	2
	MRB02	85.0408	80.9198	76.7152	88.0654	77.5613	2
	ZXZ15(α)	85.0408	80.9198	76.7152	88.0654	77.5613	2
	VMH16	86.2602	83.1861	79.1152	88.9225	79.3903	1
	RH17	84.7331	80.4198	76.3152	87.8154	77.0997	4
	AKO18a	84.3305	80.2688	75.3495	87.5048	76.4957	5
	AKO18o	86.2602	83.1861	79.1152	88.9225	79.3903	1
	RH18	85.0408	80.9198	76.7152	88.0654	77.5613	2
**More than two *fpfs*-Matrices**	SM13(w,α)	84.9307	81.0648	76.5697	87.9573	77.3960	6
	NKY17(λ)	85.2384	80.8994	76.9818	88.2803	77.8575	2
	Z14/2	85.0408	80.9198	76.7152	88.0654	77.5613	3
	MR13	85.0408	80.9198	76.7152	88.0654	77.5613	3
	MR13/2	84.9345	81.1394	76.6707	87.9455	77.4017	5
	RS16	85.0408	80.9198	76.7152	88.0654	77.5613	3
	AT18(λ)	85.0408	80.9198	76.7152	88.0654	77.5613	3
	P18	84.7331	80.4198	76.3152	87.8154	77.0997	7
	A19/2(R)	86.2602	83.1861	79.1152	88.9225	79.3903	1
	SS19/2	84.9383	80.6420	76.5818	87.9987	77.4074	4
	SS19/3	85.0408	80.7739	76.8040	88.0820	77.5613	3
	SS19/4	84.7293	80.7125	76.2141	87.7955	77.0940	8
	S19/5(w).	85.0408	80.9198	76.7152	88.0654	77.5613	3
	ZCW19(δ,θ)	85.0408	80.9198	76.7152	88.0654	77.5613	3

**Table 11:** Performance comparisons of algorithms on the Libras Movement.

Single *fpfs*-Matrices	Algorithms	Acc	Pre	Rec	Spe	F1-score	Ranking
CXL13(λ)	96.9556	79.8555	77.3067	98.3693	77.1667	6
WQ14(κ)	96.9926	80.0974	77.5467	98.3892	77.4444	4
YHX14(α,β)	97.2000	81.5463	79.1333	98.4999	79.0000	2
DC15(α)	96.9481	79.7084	77.2267	98.3654	77.1111	7
ZZ19(λ,γ)	96.9556	79.8095	77.2933	98.3693	77.1667	6
CEC11	97.0074	80.0103	77.6400	98.3970	77.5556	2
MBR01	96.9704	79.9432	77.4000	98.3769	77.2778	5
G17(R)	97.0000	80.0271	77.5867	98.3931	77.5000	3
LQP17(w)	97.0000	80.0271	77.5867	98.3931	77.5000	3
KM11(𝐼𝑛))	96.9926	80.0259	77.5600	98.3892	77.4444	4
LL18(𝝀)	96.9926	80.0395	77.5467	98.3892	77.4444	4
A19(R,w,λ1,λ2,λ3,λ4,λ5)	97.2296	81.7945	79.3600	98.5158	79.2222	1
**Double *fpfs*-Matrices**	CXL13/2(λ)	96.9556	79.8555	77.3067	98.3693	77.1667	2
	HG13	97.0000	80.0271	77.5867	98.3931	77.5000	3
	MRB02	97.0000	80.0271	77.5867	98.3931	77.5000	3
	ZXZ15(α)	97.0000	80.0271	77.5867	98.3931	77.5000	3
	VMH16	97.3259	82.4116	80.0667	98.5675	79.9444	5
	RH17	97.0074	80.0103	77.6400	98.3970	77.5556	4
	AKO18a	96.9185	79.8717	76.9867	98.3493	76.8889	5
	AKO18o	97.3259	82.4116	80.0667	98.5675	79.9444	1
	RH18	97.0000	80.0271	77.5867	98.3931	77.5000	3
**More than two *fpfs*-Matrices**	SM13(w,α)	96.9778	79.9620	77.4267	98.3813	77.3333	7
	NKY17(λ)	96.9704	79.9432	77.4000	98.3769	77.2778	8
	Z14/2	97.0000	80.0271	77.5867	98.3931	77.5000	5
	MR13	97.0000	80.0271	77.5867	98.3931	77.5000	5
	MR13/2	97.0370	80.3105	77.8933	98.4126	77.7778	2
	RS16	97.0000	80.0271	77.5867	98.3931	77.5000	5
	AT18(λ)	97.0000	80.0996	77.5867	98.3932	77.5000	5
	P18	97.0074	80.0103	77.6400	98.3970	77.5556	4
	A19/2(R)	97.3259	82.4116	80.0667	98.5675	79.9444	1
	SS19/2	97.0000	80.0996	77.5867	98.3932	77.5000	5
	SS19/3	96.9926	80.0799	77.5467	98.3892	77.4444	6
	SS19/4	97.0296	80.2432	77.8800	98.4085	77.7222	3
	S19/5(w).	97.0000	80.0271	77.5867	98.3931	77.5000	5
	ZCW19(δ,θ)	97.0000	80.0271	77.5867	98.3931	77.5000	5

**Table 12:** Performance comparisons of algorithms on the Teaching dataset.

Single *fpfs*-Matrices	Algorithms	Acc	Pre	Rec	Spe	F1-score	Ranking
CXL13(λ)	76.9405	66.1145	65.1973	82.6825	65.4108	6
WQ14(κ)	77.2961	66.5722	65.7428	82.9524	65.9441	4
YHX14(α,β)	78.0043	67.8592	66.8094	83.4959	67.0065	2
DC15(α)	77.0265	66.2413	65.3306	82.7492	65.5398	5
ZZ19(λ,γ)	77.0265	66.2413	65.3306	82.7492	65.5398	5
CEC11	77.2961	66.5722	65.7428	82.9524	65.9441	4
MBR01	77.5599	67.0105	66.1306	83.1492	66.3398	3
G17(R)	77.2961	66.5722	65.7428	82.9524	65.9441	4
LQP17(w)	77.2961	66.5722	65.7428	82.9524	65.9441	4
KM11(𝐼𝑛))	77.2961	66.5722	65.7428	82.9524	65.9441	4
LL18(𝝀)	77.2961	66.5722	65.7428	82.9524	65.9441	4
A19(R,w,λ1,λ2,λ3,λ4,λ5)	78.0932	68.0391	66.9428	83.5626	67.1398	1
**Double *fpfs*-Matrices**	CXL13/2(λ)	76.9405	66.1145	65.1973	82.6825	65.4108	4
	HG13	77.2961	66.5722	65.7428	82.9524	65.9441	3
	MRB02	77.2961	66.5722	65.7428	82.9524	65.9441	3
	ZXZ15(α)	77.2961	66.5722	65.7428	82.9524	65.9441	3
	VMH16	77.9154	67.7050	66.6761	83.4292	66.8731	1
	RH17	77.2961	66.5722	65.7428	82.9524	65.9441	3
	AKO18a	77.2989	66.8443	65.7724	82.9485	65.9484	2
	AKO18o	77.9154	67.7050	66.6761	83.4292	66.8731	1
	RH18	77.2961	66.5722	65.7428	82.9524	65.9441	3
**More than two *fpfs*-Matrices**	SM13(w,α)	77.2961	66.5722	65.7428	82.9524	65.9441	4
	NKY17(λ)	77.5599	67.0105	66.1306	83.1492	66.3398	2
	Z14/2	77.2961	66.5722	65.7428	82.9524	65.9441	4
	MR13	77.2961	66.5722	65.7428	82.9524	65.9441	4
	MR13/2	77.3849	66.7164	65.8761	83.0190	66.0774	3
	RS16	77.2961	66.5722	65.7428	82.9524	65.9441	4
	AT18(λ)	77.2961	66.5722	65.7428	82.9524	65.9441	4
	P18	77.2961	66.5722	65.7428	82.9524	65.9441	4
	A19/2(R)	77.9154	67.7050	66.6761	83.4292	66.8731	1
	SS19/2	77.2961	66.5722	65.7428	82.9524	65.9441	4
	SS19/3	77.2961	66.5722	65.7428	82.9524	65.9441	4
	SS19/4	77.3849	66.7164	65.8761	83.0190	66.0774	3
	S19/5(w).	77.2961	66.5722	65.7428	82.9524	65.9441	4
	ZCW19(δ,θ)	77.2961	66.5722	65.7428	82.9524	65.9441	4

**Table 13:** Performance comparisons of algorithms on the Ionosphere dataset.

Single *fpfs*-Matrices	Algorithms	Acc	Pre	Rec	Spe	F1-score	Ranking
CXL13(λ)	87.9702	85.9995	97.2444	71.3723	91.2350	10
WQ14(κ)	88.5392	86.3473	97.7778	72.0062	91.6607	3
YHX14(α,β)	90.1907	87.9623	98.4000	75.4892	92.8325	2
DC15(α)	88.1400	86.2187	97.2444	71.8462	91.3509	8
ZZ19(λ,γ)	87.6266	85.6875	97.1556	70.5662	91.0111	11
CEC11	88.3686	86.2150	97.6889	71.6862	91.5394	5
MBR01	88.0290	85.9595	97.5111	71.0523	91.2988	9
G17(R)	88.4258	86.2742	97.6889	71.8462	91.5750	4
LQP17(w)	88.4258	86.2742	97.6889	71.8462	91.5750	4
KM11(𝐼𝑛))	88.3678	86.3817	97.4222	72.1600	91.5202	6
LL18(𝝀)	88.3107	86.0800	97.7778	71.3662	91.5086	7
A19(R,w,λ1,λ2,λ3,λ4,λ5)	90.9875	88.9734	98.4000	77.6923	93.3852	1
**Double *fpfs*-Matrices**	CXL13/2(λ)	87.9702	85.9995	97.2444	71.3723	91.2350	5
	HG13	88.4258	86.2742	97.6889	71.8462	91.5750	2
	MRB02	88.4258	86.2742	97.6889	71.8462	91.5750	2
	ZXZ15(α)	88.4258	86.2742	97.6889	71.8462	91.5750	2
	VMH16	91.1630	89.2811	98.2222	78.5046	93.4760	1
	RH17	88.3686	86.2150	97.6889	71.6862	91.5394	3
	AKO18a	88.1408	86.1789	97.3333	71.6862	91.3665	4
	AKO18o	91.1630	89.2811	98.2222	78.5046	93.4760	1
	RH18	88.4258	86.2742	97.6889	71.8462	91.5750	2
**More than two *fpfs-*Matrices**	SM13(w,α)	88.1964	85.9991	97.6889	71.2062	91.4245	7
	NKY17(λ)	88.0290	85.9595	97.5111	71.0523	91.2988	8
	Z14/2	88.4258	86.2742	97.6889	71.8462	91.5750	3
	MR13	88.4258	86.2742	97.6889	71.8462	91.5750	3
	MR13/2	88.3678	86.1381	97.8667	71.3600	91.5666	4
	RS16	88.4258	86.2742	97.6889	71.8462	91.5750	3
	AT18(λ)	88.4258	86.2742	97.6889	71.8462	91.5750	3
	P18	88.3686	86.2150	97.6889	71.6862	91.5394	5
	A19/2(R)	91.1630	89.2811	98.2222	78.5046	93.4760	1
	SS19/2	88.3686	86.2637	97.6000	71.8462	91.5303	6
	SS19/3	88.5392	86.4751	97.6000	72.3200	91.6478	2
	SS19/4	88.1972	86.0988	97.6000	71.3600	91.4255	7
	S19/5(w).	88.4258	86.2742	97.6889	71.8462	91.5750	3
	ZCW19(δ,θ)	88.4258	86.2742	97.6889	71.8462	91.5750	3

**Table 14:** Performance comparisons of algorithms on the Whosalers dataset.

Single *fpfs*-Matrices	Algorithms	Acc	Pre	Rec	Spe	F1-score	Ranking
CXL13(λ)	69.9091	32.0641	32.1161	65.55921	54.8636	9
WQ14(κ)	70.0303	33.2414	32.5892	65.9406	55.0454	6
YHX14(α,β)	70.4242	33.3163	33.1314	65.86009	55.6363	1
DC15(α)	70.0606	32.8172	32.6976	65.83018	55.0909	5
ZZ19(λ,γ)	69.9394	32.5119	32.5008	65.71994	54.9090	8
CEC11	70.1515	33.3109	32.6238	66.04642	55.2272	4
MBR01	70.2424	33.0922	32.4225	65.95886	55.3636	3
G17(R)	70.1515	33.3109	32.6238	66.04642	55.2272	4
LQP17(w)	70.1515	33.3109	32.6238	66.04642	55.2272	4
KM11(𝐼𝑛))	70	33.1103	32.7666	66.06458	55	7
LL18(𝝀)	69.9090	33.1822	32.4548	66.01832	54.8636	9
A19(R,w,λ1,λ2,λ3,λ4,λ5)	70.3636	33.1482	33.0532	65.64021	55.5454	2
**Double *fpfs*-Matrices**	CXL13/2(λ)	69.9091	32.0641	32.1161	65.55921	54.8636	4
	HG13	70.1515	33.3109	32.6238	66.04642	55.2273	2
	MRB02	70.1515	33.3109	32.6238	66.04642	55.2273	2
	ZXZ15(α)	70.1515	33.3109	32.6238	66.04642	55.2273	2
	VMH16	70.5151	33.6322	33.4485	65.98761	55.7727	1
	RH17	70.1515	33.3109	32.6238	66.04642	55.2273	2
	AKO18a	70	32.0723	31.9901	65.57787	55	3
	AKO18o	70.51515	33.6322	33.4484	65.98761	55.7727	1
	RH18	70.15152	33.3109	32.6238	66.04642	55.2273	2
**More than two *fpfs*-Matrices**	SM13(w,α)	69.9394	33.1636	32.6562	66.1404	54.9091	5
	NKY17(λ)	70.2424	33.0922	32.4225	65.95886	55.3636	2
	Z14/2	70.1515	33.3109	32.6238	66.04642	55.2273	3
	MR13	70.1515	33.3109	32.6238	66.04642	55.2273	3
	MR13/2	69.8788	33.1315	32.2403	65.73888	54.8182	6
	RS16	70.1515	33.3109	32.6238	66.04642	55.2273	3
	AT18(λ)	70.1515	33.3109	32.6238	66.04642	55.2273	3
	P18	70.1515	33.3109	32.6238	66.04642	55.2273	3
	A19/2(R)	70.5151	33.6322	33.4485	65.98761	55.7728	1
	SS19/2	70.1515	33.3109	32.6238	66.04642	55.2273	3
	SS19/3	70.1515	33.3109	32.6238	66.04642	55.2273	3
	SS19/4	70	33.1366	32.3122	65.59	55	4
	S19/5(w).	70.15152	33.31088	32.6238	66.04642	55.2273	3
	ZCW19(δ,θ)	70.15152	33.31088	32.6238	66.04642	55.2273	3

**Table 15:** Performance comparisons of algorithms on the Glass dataset.

Single *fpfs*-Matrices	Algorithms	Acc	Pre	Rec	Spe	F1-score	Ranking
CXL13(λ)	87.9719	61.4511	58.3238	91.6998	63.9158	10
WQ14(κ)	88.6555	64.9785	62.2627	92.1690	65.9667	5
YHX14(α,β)	90.4665	69.5604	68.1317	93.4853	71.3997	2
DC15(α)	88.1587	62.0427	59.2131	91.8283	64.4761	9
ZZ19(λ,γ)	88.1897	62.2378	59.2666	91.8459	64.5692	8
CEC11	88.5315	64.4263	61.4511	92.0929	65.5946	7
MBR01	89.1878	66.7765	64.7746	92.5778	67.5636	3
G17(R)	88.5625	64.3303	61.5484	92.1033	65.6877	6
LQP17(w)	88.5625	64.3303	61.5484	92.1033	65.6877	6
KM11(𝐼𝑛))	88.6866	64.6516	62.0119	92.1894	66.0598	5
LL18(𝝀)	88.7183	64.8068	62.0071	92.2199	66.1550	4
A19(R,w,λ1,λ2,λ3,λ4,λ5)	90.6519	69.9312	68.9353	93.6705	71.9557	1
**Double *fpfs*-Matrices**	CXL13/2(λ)	87.9719	61.4511	58.3238	91.6998	63.9158	3
	HG13	88.5625	64.3303	61.5484	92.1033	65.6877	2
	MRB02	88.5625	64.3303	61.5484	92.1033	65.6877	2
	ZXZ15(α)	88.5625	64.3303	61.5484	92.1033	65.6877	2
	VMH16	90.8401	70.5550	69.2027	93.8122	72.5204	1
	RH17	88.5315	64.4263	61.4511	92.0929	65.5946	2
	AKO18a	87.9402	61.4008	57.3738	91.6721	63.8206	4
	AKO18o	90.8401	70.5550	69.2027	93.8122	72.5204	1
	RH18	88.5625	64.3303	61.5484	92.1033	65.6877	2
**More than two *fpfs*-Matrices**	SM13(w,α)	88.6563	64.4022	61.6150	92.1723	65.9689	5
	NKY17(λ)	89.1878	66.7765	64.7746	92.5778	67.5636	2
	Z14/2	88.5625	64.3303	61.5484	92.1033	65.6877	8
	MR13	88.5625	64.3303	61.5484	92.1033	65.6877	8
	MR13/2	88.7183	65.0309	62.4857	92.2281	66.1550	4
	RS16	88.5625	64.3303	61.5484	92.1033	65.6877	8
	AT18(λ)	88.5935	64.5208	61.8817	92.1213	65.7807	7
	P18	88.5315	64.4263	61.4511	92.0929	65.5946	9
	A19/2(R)	90.8401	70.5550	69.2027	93.8122	72.5204	1
	SS19/2	88.5935	64.5208	61.8817	92.1213	65.7807	7
	SS19/3	88.6238	64.9430	61.9230	92.1271	65.8715	6
	SS19/4	88.8128	65.1590	63.6214	92.3303	66.4385	3
	S19/5(w).	88.5625	64.3303	61.5484	92.1033	65.6877	8
	ZCW19(δ,θ)	88.5625	64.3303	61.5484	92.1033	65.6877	8

**Table 16:** Performance comparisons of algorithms on the Ecoli dataset.

Single *fpfs*-Matrices	Algorithms	Acc	Pre	Rec	Spe	F1-score	Ranking
CXL13(λ)	93.9244	66.3831	64.0791	96.1818	77.7919	9
WQ14(κ)	94.1341	67.3096	65.5277	96.3195	78.5654	4
YHX14(α,β)	94.2466	69.0967	65.8851	96.3314	79.1062	2
DC15(α)	93.9093	66.4283	64.2077	96.1739	77.7313	10
ZZ19(λ,γ)	93.9611	66.6126	64.3791	96.2049	77.9104	8
CEC11	94.1322	67.2772	65.6325	96.3182	78.5662	3
MBR01	94.0766	67.4049	64.9964	96.2746	78.3906	6
G17(R)	94.1173	67.2557	65.4896	96.3095	78.5065	5
LQP17(w)	94.1173	67.2557	65.4896	96.3095	78.5065	5
KM11(𝐼𝑛))	94.0325	67.0181	65.0261	96.2540	78.2072	7
LL18(𝝀)	94.1021	67.0496	65.2229	96.3011	78.4460	6
A19(R,w,λ1,λ2,λ3,λ4,λ5)	94.3646	69.1964	66.2008	96.4091	79.4047	1
**Double *fpfs*-Matrices**	CXL13/2(λ)	93.9244	66.3831	64.0791	96.1818	77.7919	4
	HG13	94.1173	67.2557	65.4896	96.3095	78.5065	3
	MRB02	94.1173	67.2557	65.4896	96.3095	78.5065	3
	ZXZ15(α)	94.1173	67.2557	65.4896	96.3095	78.5065	3
	VMH16	94.3828	69.7985	66.5500	96.4186	79.5803	1
	RH17	94.1322	67.2772	65.6325	96.3182	78.5662	2
	AKO18a	93.7742	65.5431	63.5887	96.0882	77.2537	5
	AKO18o	94.3828	69.7985	66.5500	96.4186	79.5803	1
	RH18	94.1173	67.2557	65.4896	96.3095	78.5065	3
**More than two *fpfs*-Matrices**	SM13(w,α)	94.0723	67.1142	65.1453	96.2785	78.3266	7
	NKY17(λ)	94.0763	67.4049	64.9964	96.2746	78.3906	5
	Z14/2	94.1173	67.2557	65.4896	96.3095	78.5065	4
	MR13	94.1173	67.2557	65.4896	96.3095	78.5065	4
	MR13/2	94.2300	67.5950	65.7406	96.3776	78.9236	2
	RS16	94.1173	67.2557	65.4896	96.3095	78.5065	4
	AT18(λ)	94.1173	67.2557	65.4896	96.3095	78.5065	4
	P18	94.1322	67.2772	65.6325	96.3182	78.5662	3
	A19/2(R)	94.3828	69.7985	66.5500	96.4186	79.5803	1
	SS19/2	94.1173	67.2557	65.4896	96.3095	78.5065	4
	SS19/3	94.0843	67.2610	65.2678	96.2880	78.3863	6
	SS19/4	94.1255	67.5582	65.2148	96.3046	78.5662	3
	S19/5(w).	94.1173	67.2557	65.4896	96.3095	78.5065	4
	ZCW19(δ,θ)	94.1173	67.2557	65.4896	96.3095	78.5065	4

In Table 7, A19 stood out for its exceptional performance with single *fpfs*-matrices. YHX14, CEC11, G17, and LQP17 also performed exceptionally well, with accuracy above 95% and high precision, recall, and F1-score values. In the double *fpfs*-matrices, HG13, MRB02, ZXZ15, and RH18 yielded the best results. The top-performing algorithms using more than two *fpfs*-matrices were ZCW19, S19/5, RS16, Z14/2, and MR13.

In Table 8, notable achievements were observed with A19, YHX14, CEC11, and WQ14, each yielding impressive F1-scores exceeding 96% when using single *fpfs*-matrices. For double *fpfs*-matrices, AKO18o and VMH16 exhibited strong performance. Furthermore, A19/2 emerged as the most effective algorithm when leveraging more than two *fpfs*-matrices.

The comparison in Table 9 manifests varied SDM algorithm performance across different numbers of *fpfs-*matrices. It is noted that, among single *fpfs*-matrices, YHX14, MBR01, and A19 show the best performance. Furthermore, when utilizing double *fpfs*-matrices, VMH16 and AKO18 appear to perform favorably. Lastly, the results suggest that SS19/4 yields the best outcome when working with more than two *fpfs*-matrices.

Table 10 shows a comprehensive overview of the performance comparisons of algorithms on the Hayes dataset. It is worth noting that YHX14 and A19 demonstrate excellent results from the single-*fpfs*-matrices. Moreover, the double *fpfs*-matrices AKO18o algorithm stands out as the top performer. When considering matrices with more than two *fpfs*-matrices, the A19/2 algorithm emerges as the most favorable choice.

Table 11 provides performance results for the Libras dataset. We can state that A19 is the best algorithm. The findings indicate that among the double *fpfs*-matrices, AKO18a yields the most favorable outcomes. Considering two or more *fpfs*-matrices, it is evident that A19/2 yields the best result for the Libras movement dataset.

In Table 12, which details the performance of the teaching dataset, Algorithm A19 produces the best results. Among the double *fpfs*-matrices, AKO18o seems to generate the most favorable outcomes. Moreover, when considering two or more *fpfs*-matrices, A19/2 delivers the best results for the teaching dataset.

Table 13 provides a detailed comparison of algorithm performance on the Ionosphere dataset. A19 shows excellent results with single *fpfs*-matrices, while the double *fpfs*-matrices AKO18o algorithm stands out as the top performer. When considering matrices with more than two *fpfs*-matrices, the A19/2 algorithm emerges as the most favorable choice.

Table 14 compares algorithm performance on the Wholesalers dataset. YHX14 excels with single *fpfs*-matrices, while AKO18o stands out with double *fpfs*-matrices. A19/2 is the top choice for matrices with more than two *fpfs*-matrices.

Table 15 presents a comprehensive comparison of algorithm performance on the Glass dataset. A19 demonstrates commendable results with single *fpfs*-matrices, while the double *fpfs*-matrices AKO18o algorithm emerges as the leading performer. When evaluating matrices with more than two *fpfs*-matrices, the A19/2 algorithm appears to be the most favorable choice.

In Table 16, from the single *fpfs*-matrices, A19 emerges as the top performer, with the highest values across most metrics, including an F1-score of 79.40474. YHX14 and CEC11 also perform well, securing second and third positions. Most algorithms maintain high specificity (above 96%) and accuracy of around 94%.

When we consider Table 17, which compares all datasets for single matrices, we observe that A19 usually yields the best result, followed by MBR01 and YHX14. In most datasets, the CXL13 algorithm produces the worst results.

**Table 17:** Ranking Single *fpfs*-matrices for ten datasets.

Dataset	*Single fpfs*-*matrices (F1-score-based rankings)*
Hayes	CEC11≺ ZZ19≺ CXL13≈DC15≺ WQ14≺ G17≈LQP17≺ LL18≺ KM11≈MBR01≈ YHX14≺A19
Ionosphere	ZZ19≺ CXL13≺MBR01≺DC15≺LL18≺KM11≺ CEC11≺G17≈ LQP17≺WQ14≺ YHX14≺A19
Libras	DC15≺ CXL13≈ZZ19≺MBR01≈WQ14≈KM11≈LL18≺G17≈ LQP17≺CEC11≺ YHX14≺A19
Parkinsons	CXL13 ≺ ZZ19 ≺ DC15 ≺ MBR01 ≺ KM11 ≺ LL18 ≺ WQ14 ≺ CEC11≺ YHX14 ≺ G17 ≈ LQP17 ≺ A19
Sonar	CXL13≺ ZZ19≈DC15≺KM11≺WQ14≺G17≈LQP17≺CEC11≺ LL18≺A19≺MBR01≺YHX14
Teaching	CXL13≺DC15≈ZZ19≺WQ14≈CEC11≈G17≈ LQP17≈KM11≈ LL18≺MBR01≺YHX14≺A19
Wine	CXL13 ≺ ZZ19 ≈ DC15 ≺ MBR01 ≺ KM11 ≺ LL18 ≈ LQP17 ≺G17≺ YHX14 ≺WQ14≺ CEC11 ≺ A19
Whosaler	LL18≈CXL13≺ ZZ19≺KM11≺WQ14≺DC15≺CEC11≈ G17≈ LQP17≺MBR01≺A19≺YHX14
Glass	CXL13≺ DC15≺ZZ19≺CEC11≺G17≈LQP17≺ KM11≈WQ14≺LL18≺MBR01≺ YHX14≺A19
Ecoli	DC15≺ CXL13≺ZZ19≺KM11≺LL18≈ MBR01≺G17≈ LQP17≺WQ14≺CEC11 ≺YHX14≺A19

When analyzing Table 18, we notice that AKO18o usually yields the best result, followed by VMH16 and RH18. In most datasets, the AKO18a algorithm produces the worst results.

**Table 18:** Ranking double *fpfs*-matrices for ten datasets.

Dataset	*Double fpfs*-*matrices (F1-score-based rankings)*
Hayes	AKO18a ≺ RH17 ≺ CXL13/2 ≺ HG13 ≈ MRB02 ≈ ZXZ15 ≈ RH18 ≺ VMH16 ≺ AKO18o
Ionosphere	CXL13/2≺ AKO18a≺RH17≺ HG13≈MRB02≈ZXZ15≈RH18≺ AKO18o≈VMH16
Libras	AKO18a ≈ VMH16 ≺ RH17 ≈ HG13 ≈ MRB02 ≈ ZXZ15 ≈ RH18 ≺ CXL13/2 ≺ AKO18o
Parkinsons	AKO18a ≺ CXL13/2 ≺ VMH16 ≈ AKO18o ≺ RH17 ≺ HG13 ≈ ZXZ15 ≈ RH18 ≈ MRB02
Sonar	AKO18a ≺ CXL13/2 ≺ HG13 ≈ MRB02 ≈ ZXZ15 ≈ RH18 ≺ RH17 ≺ VMH16 ≈ AKO18o
Teaching	CXL13/2 ≺ HG13 ≈ MBR02 ≈ ZXZ15 ≈ RH17 ≈ RH18 ≺ AKO18a ≺ VMH16 ≈ AKO18o
Wine	AKO18a ≈ CXL13/2 ≺ ZXZ15 ≈ RH18 ≈ MRB02 ≈ HG13 ≺ RH17 ≺ VMH16 ≈ AKO18o
Whosaler	CXL13/2≺ AKO18a≺RH17≈HG13≈MRB02≈ZXZ15≈RH18≺VMH16≈ AKO18o
Glass	AKO18a≺CXL13/2≺RH17≈HG13≈MRB02≈ZXZ15≈RH18≺VMH16≈ AKO18o
Ecoli	AKO18a≺ CXL13/2≺HG13≈ MRB02≈ZXZ15≈MRB02≈RH18≺VMH16≈ AKO18o

In Table 19, A19/2 shows the best result. In three of 10 datasets, the NKY17 algorithm generates the worst results.

**Table 19:** Sorting more than two *fpfs*-matrices for ten datasets.

Dataset	*More than two fpfs-matrices (F1-score-based rankings)*
Hayes	SS19/4≺P18≺ SM13≺ MR13/2≈SS19/2≺Z14/2 ≈MR13≈RS16≈AT18≈SS19/3≈S19/5≈ZCW19≺NKY17≺A19/2
Ionosphere	NKY17≺SM13≈ SS19/4≺ SS19/2≺P18≺MR13/2≺Z14/2≈MR13≈RS16≈AT18≈S19/5≈ZCW19≺ SS19/3≺A19/2
Libras	NKY17≺SM13≺ SS19/3≺ Z14/2≈MR13≈RS16 ≈AT18≈SS19/2≈S19/5≈ZCW19≺P18≺SS19/4≺MR13/2≺A19/2
Parkinsons	A19/2 ≺ NKY17 ≺ SM13 ≈ SS19/3 ≈ SS19/4 ≺ P18 ≺ SS19/2 ≈AT18≺ MR13/2 ≺ S19/5≈ZCW19 ≈RS16≈ Z14/2 ≈MR13
Sonar	SS19/3≺Z14/2≈ MR13≈ RS16≈S19/5≈ZCW19 ≺P18≈AT18≈SS19/2≺SM13≺MR13/2≺A19/2≺NKY17≺SS19/4
Teaching	SM13≈Z14/2≈MR13≈RS16≈ AT18≈P18≈SS19/2≈SS19/3≈S19/5≈ZCW19≺MR13/2 ≈SS19/4≈A19/2
Wine	NKY17 ≺ SM13 ≈ Z14/2 ≈MR13 ≈RS16≈AT18≈SS19/2≈SS19/3 ≈SS19/5≈ ZCW19 ≺MR13/2 ≈SS19/4≺P18≺A19/2
Whosaler	MR13/2≺SM13≺ SS19/4≺ Z14/2≈MR13≈RS16≈AT18≈P18≈SS19/2≈SS19/3≈S19/5≈ZCW19≺ NKY13≺A19/2
Glass	P18≺S19/5≈ZCW19≈RS16≈Z14/2≈MR13≈S19/2≈AT18≺SS19/3≺SM13≺MR13/2≺ SS19/4≺NKY17≺A19/2
Ecoli	SM13≺SS19/2≺NKY17≺Z14/2≈MR13≈RS16≈ AT18≈ SS19/2≈ S19/5≈ ZCW19≺SS19/4≈ PS18≺ MR13/2≺A19/2

Our study considered datasets from real-world application domains, including Medicine, Chemistry, Acoustics, and Artificial Intelligence. Our analysis based on Tables 17–19 identified the best-performing SDM methods for each dataset as follows:

- Hayes-Roth (Artificial Intelligence): A19, AKO18o, A19/2
- Ionosphere (Radio-Frequency Analysis): A19, VMH16, A19/2
- Libras (Biomechanics): A19, AKO18o, A19/2
- Parkinsons[sic] (Medical): A19, MRB02, MR13
- Sonar (Acoustics): YHX14, AKO18o, SS19/4
- Teaching Assistant Evaluation (Educational Sciences): A19, AKO18o, A19/2
- Wine (Chemistry): CEC11, AKO18o, A19/2
- Wholesaler (Business Analytics): YHX14, AKO18o, A19/2
- Glass (Forensic Sciences): A19, AKO18o, A19/2
- Ecoli (Biology): A19, AKO18o, A19/2

The success of A19 on the Wine dataset is specific to this dataset; it cannot be directly generalized to all problems in the field of Chemistry. Each dataset has different dynamics and features tailored to its field. Therefore, the superior performance of A19 on the Wine dataset does not imply that it will be generally successful in such applications. Consequently, the performance of specific algorithms can be evaluated more accurately by considering the unique structure of each dataset.

## 6. Statistical Evaluation

This section employs the corrected Friedman test [76] and the Nemenyi post hoc test [77], following the recommendations of [78], to assess the statistical significance of overall differences in performance across five performance metrics. The Friedman nonparametric test is used for multiple-hypothesis testing and provides a performance-based ranking of algorithms for each dataset. It assigns average ranks when algorithms are tied, with 1 indicating the best-performing algorithm, 2 the second-best, and so on. A rank of 35 represents the worst algorithm. It evaluates the Friedman statistic according to the, $\overset{\text{2}}{\underset{F}{\;\chi }}$ distribution with k − 1 degrees of freedom, where *k* denotes the number of algorithms. If a statistically significant difference in performance is observed, a post hoc test is used to determine which difference corresponds to which algorithm. The Nemenyi test is a commonly used post hoc test for comparing different classifiers. In this test, if the average ranks of two algorithms differ by more than the critical distance, their performance is significantly different. Initially, we calculate the average rank of each algorithm considered in our experiments, where the total number of algorithms k =35 and the number of datasets N = 10.

**Fig 1:**
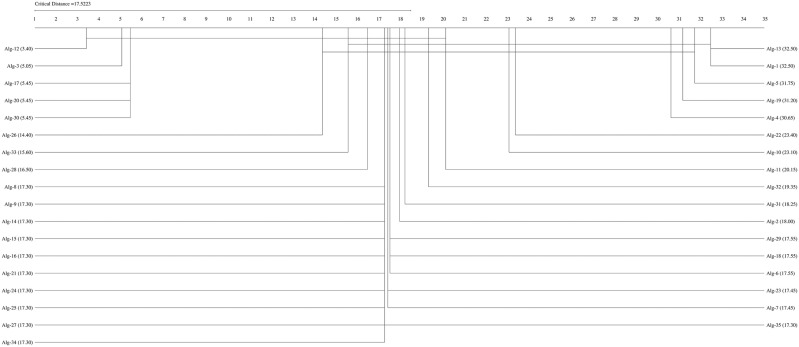
The Nemenyi diagrams (at 0.05 significance levels) of the mean Acc performances of 35 SDM methods.

**Fig 2:**
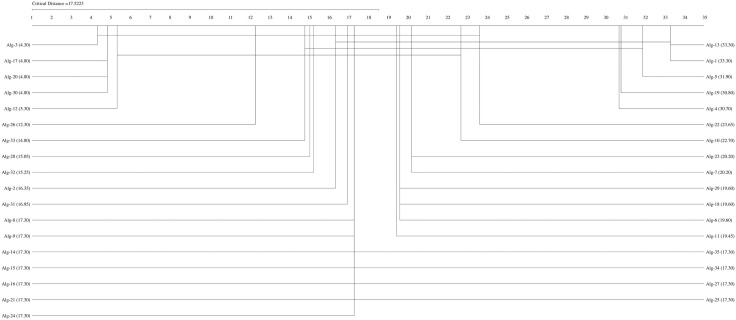
The Nemenyi diagrams (at 0.05 significance levels) of the mean Pre performances of 35 SDM methods.

**Fig 3:**
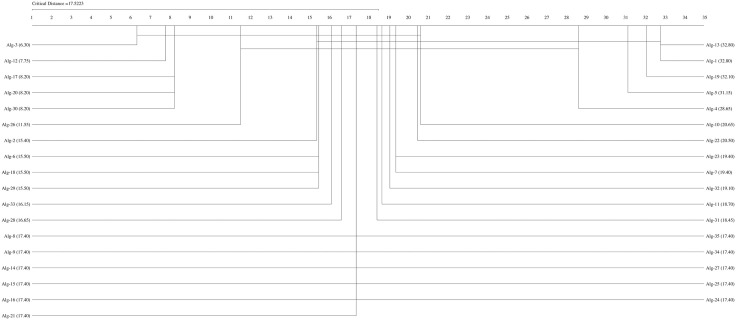
The Nemenyi diagrams (at 0.05 significance levels) of the mean Rec performances of 35 SDM methods.

**Fig 4:**
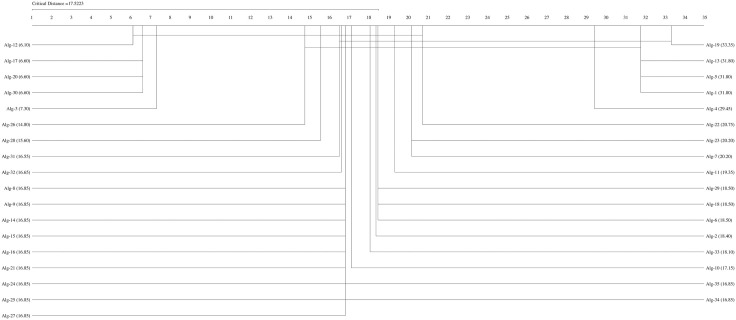
The Nemenyi diagrams (at 0.05 significance levels) of the mean Spe performances of 35 SDM methods.

**Fig 5:**
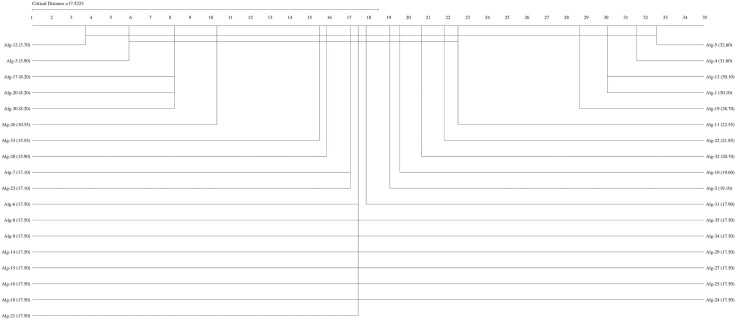
The Nemenyi diagrams (at 0.05 significance levels) of the mean F1-score performances of 35 SDM methods.

**Fig 6:**
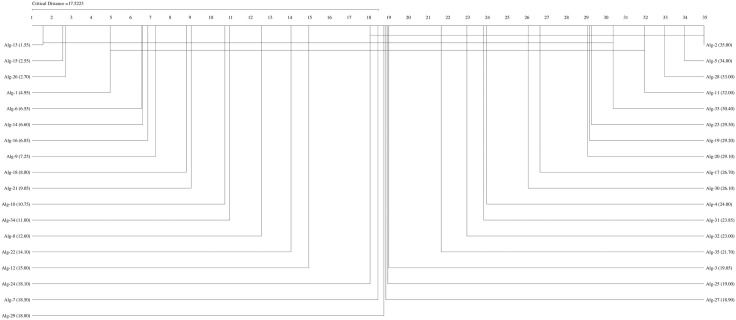
The Nemenyi diagrams (at 0.05 significance levels) of the Running Times performances of 35 SDM methods.

If the Friedman test statistic values for the accuracy, precision, recall, specificity, and F1 score values are $\overset{\text{2}}{\underset{F}{\;\chi }}=\text{1}\text{9}\text{4}.\text{3}\text{2}\overset{\text{2}}{\underset{F}{\;\chi }}=\text{ 204}.\text{18},$ $\overset{\text{2}}{\underset{F}{\;\chi }}=\text{1}\text{6}\text{2}.\text{3}\text{4}$, $\overset{\text{2}}{\underset{F}{\;\chi }}=\;\text{1}\text{5}\text{9}.\text{4}\text{2}$, and $\overset{\text{2}}{\underset{F}{\;\chi }}=\;\text{1}\text{9}\text{2}.\text{0}\text{1}$, respectively, with 35 (k−1) degrees of freedom and the critical value for the Friedman test (Friedman, 1940) given for k = 35 and N = 10 is 9.49 at a significance level of α = 0.05, we can conclude that the accuracy (194.32>9.49), precision (204.18,>9.49), recall (162.34>9.49), F1-score (192.01>9.49), and specificity (159.42>9.49) values of the studied methods are significantly different. We can proceed with a post hoc test after rejecting the null hypothesis. The Nemenyi test [77] can be used when comparing all classifiers [78]. The Nemenyi diagrams are presented in Figs 1–6.

Figs 1–5 depict the top five SDM methods, which exhibit varying rankings across Acc, Spe, F1-score, and Rec performance. These methods are Alg-12 (A19), Alg-3 (YHX14), Alg-17 (VMH16), Alg-20 (AKO18o), and Alg-30 (A19/2).

In machine learning, relying solely on accuracy can be misleading, especially when precision and recall are inversely related. To address this, we used 10 diverse UCI datasets to establish a more reliable performance baseline. The F1-score was chosen as the main ranking metric because it provides a harmonic mean of precision and recall, ensuring that neither false positives nor false negatives are overlooked. This is particularly important in real-world situations where datasets may be imbalanced and a balanced evaluation of predictive quality is necessary.

To assess the importance of these rankings, we conducted a thorough two-stage statistical analysis using the Friedman and Nemenyi tests. First, the Friedman test was used to assess significant differences across the 35 SDM algorithms across all datasets. After rejecting the null hypothesis (which suggested that the performance differences were due to chance), we conducted the Nemenyi post-hoc test. This comparison examined pairs of algorithms to identify specific performance groups and notable gaps between methods.

Based on the improved F1-score analysis and the statistical results, Alg-12 (A19) proved to be the most reliable algorithm, followed by Alg-3 (YHX14). Other top-performing methods, such as Alg-30 (A19/2), Alg-20 (AKO18o), Alg-17 (VMH16), and Alg-26 (MR13/2), shared third place. The close match between our simulation-based F1 rankings and the Nemenyi test outcomes strongly supports the validity and broad applicability of the FPFS-CMC framework across different data scenarios.

## 7. Computational Complexity Analysis

This section provides a formal analysis of the computational complexity of the evaluated SDM methods using Big O notation. To ensure a clear understanding of how these complexities are derived, we first present a step-by-step analytical breakdown of G17(R) as a representative case study, examining its performance in relation to the number of samples (m) and attributes (n).

The computational efficiency of the G17(R) algorithm is primarily determined by the dimensions of the input *fpfs*-matrix $\left[a_{ij}\right]$, where m represents the number of samples and n represents the number of parameters. In Step 1, the construction or initialization of the m× n matrix requires processing every entry, resulting in a complexity of O*(*mn). While Step 2 is a simple index identification process of O(n), the subsequent operations in Steps 3 and 4 involve summations over the index set R and the entire parameter set for each of the m−1 samples. In the worst-case scenario, where R includes all parameters, these steps require (m−1)×n operations, maintaining the dominant complexity at O*(*mn). The final stages (Steps 5–7), which include finding the maximum value in the $\left[b_{i\text{1}}\right]$ vector, constructing the score matrix, and attaining the decision set, are linear operations relative only to the number of samples m. Consequently, when summing all computational costs, the higher-order term O*(*mn) prevails, leading to a total time complexity of O*(*mn)*.*

The memory overhead of the G17(R) algorithm is governed by the data structures required to store membership degrees and intermediate results. The most significant memory consumption occurs in Step 1, where the *fpfs*-matrix $\left[a_{ij}\right]$ is stored, requiring O*(*mn) space to hold the membership values for m samples across n parameters. The auxiliary vectors generated in the following steps, such as $\left[b_{i\text{1}}\right]$, $\left[c_{i\text{1}}\right]$, and the score matrix $\left[s_{i\text{1}}\right]$, are (m−1)×1 column vectors, thus requiring only O*(*m) additional space. Furthermore, the index sets R, V, and U consume negligible memory proportional to n or m. Since the storage of the initial *fpfs*-matrix remains the most resource-intensive component, the overall space complexity of the algorithm is formally defined as O*(*mn).

The formal derivation of the G17(R) algorithm serves as a template for analyzing the complexity of the other methods examined in this study. By following a similar matrix-based procedure, including fuzzification, parameter-based summation, and score function evaluation, the time and space complexities of the remaining 34 SDM algorithms can be easily determined. These findings are summarized in Table 20, which shows the Big O notation for each method and highlights their scalability across various real-world problem sizes.

**Table 20:** Computational complexity of the SDM methods.

No	Algorithm Abbreviation	Big O Notation	No	Algorithm Abbreviation	Big O Notation
**1**	CXL13(λ)	O(mn)	**19**	AKO18a	$O(mn_{\text{1}}n_{\text{2}})$
**2**	WQ14(κ)	$O(m^{\text{4}}+m^{\text{2}}n)$	**20**	AKO18o	$O(mn_{\text{1}}n_{\text{2}})$
**3**	YHX14(α,β)	O(mn)	**21**	RH18	O(mn)
**4**	DC15(α)	$O(mn^{\text{2}})$	**22**	SM13(w,α)	O(mnt)
**5**	ZZ19(λ,γ)	$O(m^{\text{2}}n)$	**23**	NKY17	$O(m^{\text{2}}n)$
**6**	CEC11,	O(mn)	**24**	Z14/2	O(mnt)
**7**	MBR01	$O(m^{\text{2}}n)$	**25**	MR13	O(mnt)
**8**	G17(R)	O(mn)	**26**	MR13/2	O(mnt)
**9**	LQP17(w)	O(mn)	**27**	RS16	O(mnt)
**10**	KM11(𝐼𝑛))	O(mn)	**28**	AT18(λ)	O(mnt)
**11**	LL18 (λ)	$O(m^{\text{2}}n)$	**29**	P18	O(mnt)
**12**	A19	O(mn)	**30**	A19/2(R)	O(mnt)
**13**	CXL13/2(λ)	O(mn)	**31**	SS19/2	O(mnt)
**14**	HG13	O(mn)	**32**	SS19/3	O(mnt)
**15**	MRB02	O(mn)	**33**	SS19/4	O(mnt)
**16**	ZXZ15(α)	O(mn)	**34**	SS19/5(w)	$O(mnt^{\text{2}})$
**17**	VMH16	$O(mn_{\text{1}}n_{\text{2}})$	**35**	ZCW19(δ,θ)	O(mnt+mtlogt)
**18**	RH17(α)	O(mn)			

Here, m denotes the row number of the *fpfs*-matrices, n, $n_{\text{1}}$, $n_{\text{2}}$ denote the column number of the *fpfs*-matrices, t denotes the number of *fpfs*-matrices.

- For the largest problems: Algorithms with O(mn) and O(mnt) complexity (e.g., CXL13, P18, MR13) are the most efficient for large-scale data due to their linear scalability.
- For Medium-Scale Problems: Algorithms with $O(mn^{\text{2}})$ or $O(m^{\text{2}}n)$ complexity (e.g., DC15, ZZ19) perform reasonably well, but time costs increase rapidly with higher dimensions.
- For Small-Scale Problems: High-complexity algorithms, such as $O(m^{\text{4}}+m^{\text{2}}n)$ (WQ14) should be employed only for very small datasets to prevent excessive computation time.
- Caution for Multiple Matrices: Algorithms with $O(mnt^{\text{2}})$ complexity (e.g., SS19/5) becomes inefficient when the number of matrices (t) is high and unsuitable for large problems.
- Special Cases: Algorithms with $O(mn_{\text{1}}n_{\text{2}})$ complexity (e.g., AKO18a, VMH16) are only practical when $n_{\text{1}}$ and $n_{\text{2}}$ They are small; otherwise, their performance deteriorates.

Therefore, SDM algorithms should be selected based on problem size (m, n, t) and available computational resources. Notably, large datasets should be chosen with linear or linear-logarithmic complexity.

## 8. Results and Discussion

The experimental results and statistical evaluations presented earlier provide a comprehensive benchmark of 35 SDM algorithms. To fully grasp the practical significance of these findings, it is important to combine the performance results with the algorithms’ structural characteristics, as classified in Table 3.

The analyses herein show a strong link between an algorithm’s “Decision Model” and its success in machine learning classification tasks. The top-performing algorithm, A19, and the highly ranked YHX14 both use aggregation-based decision models. According to Table 3, A19 is based on an “indices set-based weighted aggregation” model, while YHX14 uses “Grey relational analysis”. The effectiveness of these models across datasets such as Hayes-Roth and Glass suggests that aggregation operators are better at capturing multidimensional uncertainty in UCI benchmark datasets than models based on spatial distance or belief functions.

The “Operation” column in Table 3 offers key insights into why some algorithms perform better. Algorithms that use weighted aggregation and comparison matrices (such as A19, MBR01, and AKO18o) tend to achieve higher F1-scores. For example, A19 uses index-set-based weighted aggregation, enabling a more detailed treatment of fuzzy parameters. MBR01 focuses on a binary comparison of alternatives, yielding more robust results in datasets with clear feature distinctions. Conversely, algorithms that rely solely on distance-based aggregation (such as CXL13) usually show lower performance metrics (Tables 17–19), indicating that simple geometric distance may not be sufficient to model complex fuzzy relationships in high-dimensional data.

A key finding of this study is the shift from “single-application” backgrounds to what can be called “general-purpose” effectiveness. Many algorithms in Table 3 were initially developed for specific “Empirical Studies,” such as medical diagnosis (XWL14), car selection (DC15), or house selection (G17). Our results show that although these algorithms were designed for niche decision problems, integrating them into the FPFS-CMC framework allows them to serve as powerful general-purpose classifiers. For instance, YHX14, created for wine quality identification, performed exceptionally well on the Sonar and Wholesaler datasets. This demonstrates the high modeling ability and versatility of *fpfs*-matrix-based SDM methods.

Choosing an optimal algorithm requires considering its parameter structures and computational costs. As shown in Table 3, some algorithms depend on complex weight matrices or multiple index sets (such as A19 and SS19/5). While these parameters can improve accuracy and F1-scores, they also increase computational complexity, as analyzed in Table 20. Algorithms with linear complexity (O(mn)), like A19 and CEC11, strike the best balance between high performance and scalability for large-scale applications. Conversely, high-complexity models such as WQ14 ($O(m^{\text{4}})$) are better suited to small datasets despite their intensive decision models.

Understanding the practical implications of these findings requires combining the performance results with the structural features and dataset characteristics. The varied structural properties of the ten UCI datasets, as shown in Table 6, are crucial in explaining the fluctuations in the performance of SDM algorithms. Three possible factors that can influence these outcomes are as follows:

- Concerning the ranking criteria and the F1-score, we used the F1-score as the main metric to evaluate the performance of SDM algorithms within the machine learning framework. Unlike accuracy, which can be misleading in certain data distributions, the F1-score offers a harmonic mean of precision and recall. Using this measure, we ensured that the rankings accurately reflect a balanced assessment of each algorithm’s ability to maintain high predictive performance for both majority and minority classes, providing a more dependable benchmark for decision-making.
- The number of features in the datasets, which varies widely from 5 to 90, significantly impacts performance. In high-dimensional datasets like Libras Movement and Sonar, algorithms that use feature fuzzification and precise parameter weighting within the FPFS-CMC framework, specifically A19 and YHX14, consistently outperform others. This indicates that these models are better at handling the increased complexity introduced by a larger number of parameters than traditional methods.
- The datasets cover a range of multi-class complexities, from binary classification to problems with up to 15 classes. While many SDM algorithms perform well on binary classification tasks, such as Parkinsons[sic] or Sonar, the performance gap widens in multi-class settings, such as Ecoli (7 classes) or Libras Movement (15 classes). In these more challenging scenarios, algorithms such as A19 and A19/2 prove to be the most effective, demonstrating greater scalability across diverse and complex decision-making environments.

In conclusion, the success of an SDM algorithm depends on the complex interaction among its mathematical operations, decision model, and the dataset’s structural dynamics. This study shows that while algorithms like A19 and YHX14 provide superior modeling capabilities, their effectiveness is confirmed by their resilience to the challenging features of real-world data. By combining qualitative classifications with quantitative benchmarks, this research provides a structured framework for selecting the most suitable SDM method based on both theoretical soundness and empirical reliability.

## 9. Conclusion

The primary goal is to comprehensively classify SDM methods, assess their effectiveness in machine learning modeling, and explore their potential applications. The classification of SDM algorithms relies on the decision model, empirical studies, mathematical operators, and the number of *fpfs*-matrices. The FPFS-CMC algorithm was employed to categorize SDM algorithms across ten real datasets from the UCI Machine Learning Repository. F1-scores ranked algorithms in machine learning, and the results were subjected to statistical analysis.

This process helped identify which SDM algorithms are most suitable for real-world applications, and which produce the best results. In this context, the proposed FPFS-CMC framework offers a systematic evaluation method that combines SDM methodologies with machine learning performance metrics, providing a practical tool for selecting appropriate algorithms based on empirical data.

The studies showed that SDM algorithms performed very well in analyzing machine learning techniques, confirmed by statistical tests. The FPFS-CMC classifier proved effective for SDM methods in machine learning. Additionally, Nemenyi post hoc testing revealed that Alg-12 (A19) performed the best, followed by Alg-3 (YHX14). Alg-30 (A19/2), Alg-20 (AKO18o), and Alg-17 (VMH16) shared the third position, with MR13/2 ranked fourth. These rankings align with the results of the simulation studies, which validated the algorithms’ performance across 10 datasets. The consistency between statistical analysis results and simulation findings further demonstrates the robustness and reliability of the proposed evaluation approach, highlighting its methodological significance for machine learning-based decision analysis.

This work focuses mainly on *fpfs*-matrices because of their ease of implementation. Since algorithmic performance varies across datasets, no single algorithm can be considered universally optimal. Evaluations must consider each dataset’s inherent features and complexity. The constraints and complexity of an algorithm may not suit every dataset. Understanding the compatibility between the algorithm and the dataset is crucial. While this can be seen as a disadvantage, it underscores the importance of dataset–algorithm compatibility and offers valuable guidance for practitioners applying SDM algorithms to real-world problems.

Furthermore, while this study establishes a comprehensive performance baseline for 35 SDM algorithms, future research will focus on conducting detailed sensitivity analyses to systematically investigate the impact of various internal parameters within the *fpfs*-matrices. Given the diverse parameter structures involved, a dedicated framework will be developed to evaluate how fluctuations in these variables influence the final decision-making outcomes, thereby adding further robustness and empirical depth to the FPFS-CMC. Future research may also extend this scope to more complex algebraic structures, such as interval-valued intuitionistic fuzzy parameterized matrices [79], which provide greater flexibility in modeling linguistic uncertainties in noise removal and image processing tasks. Incorporating advanced multi-criteria group decision-making (MCGDM) frameworks, especially those using N-soft set models and their parameter-reduction techniques, offers a promising path to improve the scalability of SDM methods [80]. Additionally, integrating hybrid expert knowledge systems, like Fermatean fuzzy soft sets [81] or Pythagorean fuzzy N-soft models combined with the PROMETHEE approach [82,83], could further enhance the robustness and ranking accuracy of the FPFS-CMC framework in complex group settings. Beyond purely mathematical extensions, exploring the behavioral and cognitive aspects of decision-making, such as the influence of emotional intelligence and proactive decision-making scales [84,85], could lead to more human-centered AI systems. Lastly, applying these combined machine learning and MCDM approaches to emerging fields, such as AI-driven personalization in smart city infrastructure and urban planning [86], remains an exciting direction for developing next-generation adaptive artificial intelligence systems.

## Declaration of generative AI and AI-assisted technologies in the writing process

During the preparation of this manuscript, the authors used Grammarly to improve the English writing. The authors have reviewed and edited the output and take full responsibility for the content of this publication.
